# Fuzzy-set qualitative comparative analysis of implementation outcomes in an integrated mental healthcare trial in South Africa

**DOI:** 10.1080/16549716.2021.1940761

**Published:** 2021-08-17

**Authors:** André Janse van Rensburg, Tasneem Kathree, Erica Breuer, One Selohilwe, Ntokozo Mntambo, Ruwayda Petrus, Arvin Bhana, Crick Lund, Lara Fairall, Inge Petersen

**Affiliations:** a Centre for Rural Health, University of KwaZulu-Natal, School of Nursing and Public Health, Durban, South Africa; b Centre for Rural Health, University of KwaZulu-Natal, Durban, South Africa; c Alan J Flisher Centre for Public Mental Health, University of Cape Town, Cape Town, South Africa; d Department of Psychology, University of KwaZulu-Natal, Durban, South Africa; e Centre for Rural Health, University of KwaZulu-Natal & South African Medical Research Council, Durban, South Africa; f Alan J Flisher Centre for Public Mental Health, University of Cape Town & Centre for Global Mental Health, King’s College London, Cape Town, South Africa; g Centre for Knowledge Translation, University of Cape Town, Cape Town, South Africa

**Keywords:** Integrated mental healthcare, health systems, primary healthcare, qualitative comparative analysis, low-to-middle income country

## Abstract

**Background:**

Integrating mental health services into primary healthcare platforms is an established health systems strategy in low-to-middle-income countries. In South Africa, this was pursued through the Programme for Improving Mental Health Care (PRIME), a multi-country initiative that relied on task-sharing as a principle implementation strategy. Towards better describing the implementation processes, qualitative comparative analysis was adopted to explore causal pathways in the intervention.

**Objective:**

This study aimed to explore factors that could have influenced key outcomes of an integrated mental healthcare intervention in South Africa.

**Methods:**

Drawing from an embedded multiple case study design, the analysis used qualitative comparative analysis. Focusing on nine PHC clinics in the Dr Kenneth Kaunda District as cases, with depression reduction scores set as outcome measures, trial data variables were modelled in a hypothetical causal process. A fuzzy-set qualitative comparative analysis was performed by 1) developing the research questions, 2) developing the fuzzy set, 3) testing necessity and 4) testing sufficiency. These steps were undertaken collaboratively among the research team.

**Results:**

The data were calibrated during several meetings among team members to gain a degree of consensus. Necessity analyses suggested that none of the causal conditions exceeded the threshold of necessity and triviality, and confirmed the inclusion of relevant variables in line with the proposed models. Sufficiency analyses produced two configurations, which were subjected to standard and specific analyses. Ultimately, the results suggested that none of the causal conditions were necessary for a reduction in depression scores to occur, while programme fidelity was identified as a sufficient condition for a reduction in scores to occur.

**Conclusions:**

The study highlights the importance of understanding implementation pathways to enable better integration of mental health services within primary healthcare in low-to-middle-income settings. It underlines the importance of programme fidelity in achieving the goals of implementation.

## Background

On the back of a more enabling global environment – exemplified by the Movement for Global Mental Health and the Sustainable Development Goals [1,2] – integration of mental health into primary healthcare (PHC) is a widely-accepted mechanism for improving access, care continuity and improved outcomes among populations in need, especially in low-to-middle-income countries (LMICs) [3,4]. This being noted, there is a growing realisation that integrated mental healthcare requires system strengthening, service collaboration and co-ordination across multiple sectors within the PHC setting [5–7].

The Programme for Improving Mental Health Care (PRIME) was a multi-country initiative that developed, implemented and evaluated packages of integrated mental healthcare in Ethiopia, India, Nepal, South Africa and Uganda [3]. The principal focus of PRIME was to generate empirical evidence for integrated mental healthcare in maternal care and PHC platforms, concentrating on affecting change in the health care organisation, the health facility, and the community. A three-stage strategy was followed. First, draft plans for integrated mental healthcare were developed. Second, a three-year implementation phase followed during which the feasibility, acceptability and impact of the intervention packages at district and sub-district level were assessed. Third, the intervention packages were scaled beyond the implementation sites to other areas within the initial implementation districts and/or to other districts across the respective countries [8].

In South Africa, the intervention package that was scaled up involved the development and integration of a collaborative care model for depression, into the integrated chronic disease management (ICDM) service delivery platform at PHC level in the Dr Kenneth Kaunda district, North West province, South Africa. A key intervention mechanism was the introduction of facility-based lay counsellors into the PHC system, who provided an evidence-based structured manualized counselling referral service under the supervision of mental health specialists [9]. Following an initial pilot evaluation of the collaborative care intervention package in four facilities [9], the intervention was scaled up to 10 additional PHC facilities in the same district, which formed the intervention arm of a pragmatic cluster randomized control trial [10], with 10 control clinics receiving care as usual. Results from the pilot evaluation indicated a clinically significant reduction in reported depression severity symptoms at 3 and 12 months in the cohort of patients identified and referred to the intervention by professional nurses, compared to the control cohort who were not identified nor referred [11]. Process data from the initial pilot sites also suggested a correlation between the amount of counselling sessions received and a reduction of depressive symptoms, as well as improved health and mental functionality at endline, and found that the intervention package was acceptable and accessible [11]. However, the effectiveness analysis of the main trial outcome, which was defined as at least a 50% improvement in PHQ-9 score at 6 months from baseline, showed no difference between the intervention and control arms [12].

It remains unclear what factors affected the causal pathway between the intervention and its implementation, its contexts, and outcomes. In addition to the ‘what’ questions raised by trial designs, there is a need to raise ‘how’ questions in order to more fully understand the implementation process [13] leading to trial outcomes. Opening the ‘black box’ of trials in order to better unpack underlying causal processes has become a central aim of complex intervention programmes [14]. Documenting processes and results that describe differences from earlier hypotheses further supports translation of the intervention to other contexts [15]. These methodological considerations have been taken up by multi-country mental health strengthening programmes, such as PRIME, that aims to develop, implement and scale-up integrated mental healthcare models, considerations that are applied in order to speed up knowledge translation of mental healthcare interventions and increase their relevance and adaptability to other contexts [16,17]. The results of the PRIME South Africa trial require further scrutiny in order to better understand what elements of the intervention process require attention in order to improve identification and referral pathways [12].

This paper reports on the assessment of process indicators that were collected alongside the trial outcome data, in order to elucidate the implementation processes that may have impacted on the negative trial outcome results. The introduction of a complex collaborative care model for depression care in a health system with substantial and persistent challenges is mired in potential difficulties. The complexity of the collaborative care model is not only seated in its programmatic components, but emerges in the interaction of the intervention with its surrounding contexts – this is a critical consideration in achieving implementation success [18]. Given this complexity, a good understanding of the implementation process is as important as knowing the outcomes – ‘the challenge of scaling up mental health services in LMIC is less one of what to implement, than one of how to implement’ [19]. Despite its centrality in LMIC health policies and plans [20], empirical evidence on the implementation of integrated primary mental healthcare into real-life contexts has been limited. Towards this end, this study aimed to elucidate the factors that influence key outcomes of a complex, integrated primary healthcare intervention in the Dr Kenneth Kaunda District Municipality, North West province, South Africa.

## Methods

### Setting

The setting for the PRIME trial in South Africa has been described elsewhere [21,22], though a brief background is presented. Following consultation between the PRIME team and the National Department of Health, it was decided to develop, implement and assess the intervention in the Dr Kenneth Kaunda District, located in South Africa’s North West Province. This decision was informed by the district being a pilot site for key PHC reforms ICDM and PHC Re-engineering, thereby providing potential leverage points for health system strengthening. Located west of the city of Johannesburg, the area has a largely urbanised population of approximately 796,823 and is dominated by the mining and agricultural industries [21]. The burden of HIV is particularly high in the district (30% adult seroprevalence), while mental, neurological, and substance use disorders are among the top reasons for outpatient health service attendance [22].

### Study design

An embedded multiple case study design was used, that enabled the exploration of differences between cases [23,24], drawing from qualitative comparative analysis (QCA) to explore which intervention characteristics had an effect on the reduction in depression scores among the study population. The analysis focused on nine intervention PHC clinics of the trial in the Dr Kenneth Kaunda District as cases.

Qualitative comparative analysis (QCA) has great potential in elucidating the causal pathways that unfold in complex interventions [25]. QCA refers to a set of analytic research tools that combine within-case and cross-cases comparisons, built on the principle of complex causality [26]. QCA assumes complex and different combinations of causal factors that lead to specific outcomes [27], framed in set-theoretic terms that explore necessary and sufficient conditions [26]. In this paper, we draw from a specific type of QCA, namely fuzzy-set QCA (fsQCA). This approach has been developed to address partial membership in sets by allowing researchers to calibrate measurements that pinpoint qualitative states indicating the degree of set inclusion or exclusion [28]. fsQCA has been used to unearth complexities of implementation research in intricate health and healthcare interventions [29,30], including to evaluate the effects of a PRIME intervention in Nepal [31]. Using the reduction of rates of depression among patients in the PRIME trial in South Africa’s North West province as an outcome measure, this paper set out to explore factors that could help explain implementation factors for future scale-up efforts.

### fsQCA procedure

#### Analysis methods

The steps of the fsQCA process was undertaken in line with various guidance documents, comprising four broad steps: 1) developing the research questions, 2) developing the fuzzy set, 3) testing necessity and 4) testing sufficiency. These steps are elaborated in several helpful texts (see, for instance [25–27],), but essentially, QCA is a set theoretic method, meaning that cases are assessed based on their membership of specific conceptual sets. This makes it possible to identify important conditions, and configurations of conditions, that might help to explain how a certain occurs [32]. Central to this process is an analysis of necessity and sufficiency, namely, considering whether empirical patterns in terms of a specific condition on its own, or in combination with other conditions, could contribute to the presence of absence of an outcome [26]. A necessary pattern of conditions is one that is always present or absent when the outcome is present or absent, while a pattern of conditions is likely to be sufficient for an outcome to occur; for such a pattern to be considered sufficient, the outcome should always appear when this condition(s) is present [32].

Following the calibration of data in order to ensure uniform and systematic analysis, the data is transformed into a truth table, essentially a list of all possible configurations of conditions that might lead to the outcome, determined by the number of conditions included in the analysis. The truth table is analysed using Boolean minimisation, meaning that when two configurations are compared and differ on one condition despite leading to the same outcome, this condition is assumed to be a redundant part of the causal process, and is eliminated from the analysis [32]. Three Boolean operations are applied using a software programme, namely [26]:

- Set intersection (also known as ‘logical AND’, symbolised by ‘*’), a logical minimisation operation applied to assess membership scores in a combination of conditions that leads to the outcome;
- Set union (also known as ‘logical OR’, symbolised by ‘+’), an operation applied to assess membership scores in alternative conditions that might lead to the outcome, and
- Set negation (also known as ‘logical NOT’, symbolised by ‘~’), an operation applied to indicate an absence of conditions in explaining the outcome

Following software-assisted minimisation using these logical operations, the possible configurations of conditions are reduced and the truth table is simplified. fsQCA software allows for ‘Standard’ and ‘Specific’ analysis, which refers to different ways of minimisation. If Standard minimisation results in findings that are too broad to allow for interpretation, Specific Analysis is run additionally, to allow for the specification of possible causal pathways, resulting in parsimonious solutions. The ultimate goal is to use this process to specify the sufficient configurations that leads to the outcome [26,32].

The first step the research team undertook in this study was to collectively formulate the central research question, namely which variables explained a reduction in depression scores among the population sample. Data were compiled in an electronic spreadsheet in matrix form, according to clinic cases (rows) and variables (columns), and imported into the fsQCA software package [33] for further analysis.

#### fsQCA data sources and measurement

The primary outcome of the fsQCA were a reduction in depression scores among PHC service users enrolled in the PRIME trial on the Patient Health Questionnaire (PHQ-9) [34]. Various independent variables were collected after the 6-month trial, related to the intervention and the contexts of the implementation, which were transformed into percentage scores for fsQCA analysis. The procedures involved in obtaining these measures were derived from routine and process evaluation records including notes and scoring done by counselling supervisors, project registers, as well as from survey results and detailed below.

The availability of counsellors and other kinds of counselling that might have been received by participants were drawn manually from supervisory notes. The rates of exposure to the Adult Primary Care (APC) mental health and Clinical Communication Skills (CCS) training sessions, as well as the amount of Group Supervision sessions attended by counsellors, were obtained from project registers reflecting the number of sessions that health workers participated in. Counselling fidelity was measured by counselling supervisors, who scored the fidelity of the counselling being done by trained counsellors according to the PRIME counsellors manual, using an adapted version of the ENhancing Assessment of Common Therapeutic factors (ENACT) rating scale [35]. The average scores obtained by counsellors per facility were turned into percentages. Counselling uptake was assessing by counting, from supervisory notes, the number of sessions that participants attended out of the possible eight sessions provided. The number of referrals were derived from capturing and counting referral forms, and transformed to percentage scores out of patient headcounts per facility. Stigma and depression symptoms were measured in a pre-post patient survey, respectively, drawing from the Mental Illness Clinicians’ Attitudes scale and the PHQ9, described more fully elsewhere [9,11,12]. Finally, the Quality of Clinic Management was drawn from the Department of Health’s Ideal Clinic assessment, a routine quality assessment conducted of PHC clinics in South Africa.

A summary of variables included in the initial analysis is described in Table 1.

**Table 1.** Description of variables

Variable	Description	Measurement tool
Quality of clinic management	Composite Ideal Clinic score per clinic for the year 2017–2018	DoH Ideal Clinic measures
Availability of counsellors	Availability of counsellors per facility	Manual assessment
Other kinds of counselling received	Percentage of trial patients who received counselling from a source other than PRIME	Manual assessment
Adult Primary Care (APC) mental health training coverage	Percentage of nurses exposed to any (one or more) APC mental health sessions. Lower score means a lower proportion of nurses exposed to at least one APC mental health session	Project registers
Clinical Communication Skills (CCS) training coverage	Percentage of nurses exposed to any (one or more) CCS mental health sessions. Lower score means a lower proportion of nurses exposed to at least one CCS mental health session	Project registers
Counselling fidelity	An average score of counsellors’ scores per facility	Project registers
Counselling uptake	Number of sessions attended by patients out of a possible eight sessions per facility	Process notes
Group supervision coverage	Average number of group supervision sessions attended by counsellors per facility	Project registers
Stigma	Higher scores mean a higher level of stigma within the clinic	Mental Illness Clinicians’ Attitudes scale
Referrals	Proportion of referrals as a percentage of the total patient headcount, per facility	Referral forms
Reduction in depression scores	The proportion of patients per facility with a 50% and more reduction in PHQ9 scores	PHQ9

Several process variables from the original data set had to be excluded, following an assessment of appropriateness (using plot graphs) among the research team. These included clinic staff complement, the presence of graduate counselling students at clinics, regular visits by the district psychologist to clinics, and the availability of counsellors during the trial period. These were excluded due to little or no differentiation in scores between the clinics – a relative degree of differentiation across clinics was warranted to draw comparative conclusions on whether these differences could account for the outcome. Measurements of individual supervision coverage were excluded due to concerns about accuracy in how the data was captured. The measurement of job strain per clinic was too closely clustered together to have a substantial impact on the model, while general health status could not logically be posited as part of the hypothetical model (see Additional File Table 2).

**Table 2.** Data calibration

Variables	Type	Anchor points
Quality of clinic management	Crisp	1 = Ideal Clinic status of Gold or Platinum 0 = Ideal Clinic status less than Gold or Platinum
Additional counselling received	Fuzzy	<25% = Low >75% = High 50% = Cross-over range
APC mental health training coverage	Fuzzy	100 = All nurses exposed 49 = Low exposure 75 = Cross-over
CCS training coverage	Fuzzy	80 = All nurses exposed 30 = Low exposure 55 = Cross-over
Counselling fidelity	Fuzzy	>70% = Good 60% = Cross-over <50% = Low
Counselling uptake	Fuzzy	0 = Low sessions 0.25 = Medium sessions 0.5 = High sessions
Group supervision coverage	Crisp	0 = Low number (<70) 1 = High number (>70)
Stigma	Fuzzy	None
Referrals	Fuzzy	None
50%<PHQ9	Fuzzy	>75% = Good 62% = Medium <50% = Low

#### Hypotheses

With depression reduction set as outcome measure, variables were inductively modelled in a hypothetical causal process, during repeated meetings between research team members. Following the configuration that was used in the process evaluation [10], namely the MRC process evaluation framework’s depiction of the flow of intervention components to key outcomes as moderated by implementation factors, mechanisms of impact, and contextual consideration [36]. This model was refined and simplified among the research team following inductive application of project and case knowledge, in line with fsQCA as a method that requires iterative, back-and-forth working between the data and prior knowledge [37].

**Figure 1.**
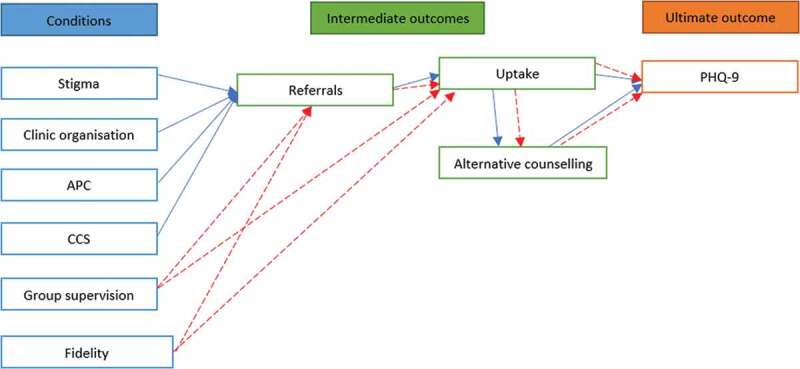
Hypothetical causal model

In this way, it was hypothesized that referrals from the nurse to the counsellor had to occur for depression scores to be reduced via the intervention, the latter can be conceptualised as the ultimate outcome, with the former as an intermediate outcome. However, given that intervention uptake, as well as seeking additional counselling following study enrolment is contingent on being referred, as well as possibly influencing the ultimate outcome, these two (intervention uptake and seeking additional counselling) were added as intermediate outcomes rather than conditions. These pathways are illustrated in Figure 1.

We hypothesized two pathways in our analysis (Figure 1). First, as shown by the blue arrows, levels of stigma in the clinic; the overall organisation of the clinic; APC and CCS training among nurses might affect the referral of patients to counselling. After referral, patients may or may not take up the intervention counselling for the 6-month duration, and they may or may not opt to take up additional forms of counselling outside of PRIME. These factors together could affect the overall reduction in depression scores.

The second pathway is illustrated by the red arrows. Here, the weight of group supervision and counselling fidelity among counsellors might have an effect on whether or not nurses refer to certain counsellors; further, group supervision and Counselling fidelity could directly affect whether or not enrolled patients would continue with the counselling, or also seek additional help, both of which could again influence rates of depression at six months.

## Results

### Calibration

Fuzzy sets were developed during research team meetings. Fuzzy logic is particularly useful in explaining complex intervention effects because it allows a graded (as opposed to strictly binary) appraisal of variable measurements. In this way, partial membership in sets can be calibrated in terms of values ranging from 0 (complete non-membership) to 1 (complete membership), with 0.5 representing a cross-over point. Accordingly, specific qualitative states can be pinpointed between these two thresholds [28]. In this way, the individual variables were each calibrated by the research team according to consensus on values that would mean that a case is fully out, fully in, and in between. Some variables did not lean towards interval grading and were included in the final set as crisp (binary) variables (Table 2).

Calibration was a collaborative, iterative process, whereby each of the variables were scrutinised by members of the research teams in terms of its relation to the outcomes of the study. In this vein, the team asked of each variable, ‘What would be a minimum score to be considered as part of the set?’, ‘What would be considered a score indicating being fully inside the set overlapping with the outcome?’, and ‘What would be a cross-over point, or middle ground, between these two anchor points?’ These qualitative judgements were largely rooted in the experience and theoretical knowledge of the team members. After applying this logic to each variable, they were calibrated using the fsQCA calibrate function. The results are presented in the Supplementary File.

### Necessity analysis

Following the ordering and calibration of the dataset, a necessity analysis was conducted (Table 3). For the main outcome of a Reduction in PHQ9 scores, two causal conditions exceeded the threshold of .90, and can therefore be considered to be necessary for the outcome to occur. These are 1) the presence of high levels of stigma in clinics (0.940298) and 2) an absence of clinical communication skills (0.981343). However, the coverage rates of these two conditions fall below 0.5, meaning that they are probably trivial (meaning they occur in most cases independent of the outcome). We attempted to find substitutable necessary conditions, whether two conditions joined by a logical ‘or’ are a necessary condition for the outcome. Based on our understanding of the intervention, the combinations Referrals + Uptake; Counsellor fidelity + Referrals; and CCS training + APC training might prove necessary for the outcome to be present. Necessity analysis were subsequently run for these combinations.

**Table 3.** Analysis of necessary conditions

Presence of outcome (Reduction in PHQ9 scores)		
Condition	**Consistency**	**Coverage**
Counselling uptake	0.585821	0.255700
~Counselling uptake	0.574627	0.538462
Group supervision	0.518657	0.278000
~Group supervision	0.481343	0.322500
Stigma	0.940298	0.363112
~Stigma	0.358209	0.466019
Quality of clinic management	0.376866	0.168333
~Quality of clinic management	0.623134	0.556667
Additional counselling	0.641791	0.442159
~ Additional counselling	0.470149	0.246575
trainingAPC training	0.835821	0.395062
~trainingAPC training	0.470149	0.378378
CCS training	0.078358	0.176471
~CCS training	0.981343	0.336748
Counsellor fidelity	0.865672	0.346786
~Counsellor fidelity	0.354478	0.411255
Referrals	0.518657	0.325527
~Referrals	0.537505	0.715956
Absence of outcome (Reduction in PHQ9 scores)		
Condition	**Consistency**	**Coverage**
Counselling uptake	0.791139	0.814332
~Counselling uptake	0.276899	0.611888
Group supervision	0.571203	0.722000
~Group supervision	0.428797	0.677500
Stigma	0.825949	0.752161
~Stigma	0.300633	0.922330
Quality of clinic management	0.789557	0.831667
~Quality of clinic management	0.210443	0.443333
Additional counselling	0.390823	0.634961
~ Additional counselling	0.656646	0.812133
trainingAPC training	0.672468	0.749559
~trainingAPC training	0.457279	0.867868
CCS training	0.180380	0.957983
~CCS training	0.844937	0.683739
Counsellor fidelity	0.784810	0.741405
~Counsellor fidelity	0.308544	0.844156
Referrals	0.517405	0.765808
~Referrals	0.544304	0.727273

As shown in Table 4, Counsellor fidelity and Referrals were a necessary combination for the presence of the outcome, but again, the coverage renders it empirically trivial as it falls below the 0.5 threshold. In terms of necessary combinations for the absence of the outcome to occur, the absence of CCS training and APC training together are suggested to be necessary for the absence of the reduction in depression scores. Ultimately, no key conditions or their combinations were found to be necessary for the presence of the reduction of PHQ9 scores. Additionally, no paradoxical results emerged, meaning no conditions were found to be necessary for the outcome to be present as well as absent. All conditions were therefore subjected to the second part of the analysis, sufficiency testing.

**Table 4.** Analysis of necessary combination conditions

Presence of outcome (reduction in PHQ9 scores)		
Condition	Consistency	Coverage
Referrals+Counselling uptake	0.805970	0.287617
Counsellor fidelity+Referrals	1.000000	0.352632
APC training+CCS training	0.835821	0.366013
Absence of outcome (Reduction in PHQ9 scores)		
Condition	**Consistency**	**Coverage**
~Referrals+~Counselling uptake	0.667722	0.691803
~Counsellor fidelity+~Referrals	0.631329	0.707447
~APC training+~CCS training	0.916139	0.700968

### Sufficiency analysis

The variables included in the study model were subjected to sufficiency analysis in fsQCA, to investigate which conditions are sufficient for the presence of the outcome to occur. This step suggested that 8 out of 512 configurations exist, with 504 being remainders to be excluded from minimisation, using a frequency threshold of 1. These eight configurations were sorted in descending order according to their raw consistency scores, which suggested that only two configurations had a consistency above the accepted threshold of 0.8. Therefore, only these two configurations had sufficient influence for PHQ9 scores to decrease. Both the proportional reduction (PRI) and symmetric consistency (SYM; a fuzzy-set equivalent of PRI) in consistency counts for these two conditions corresponded with the raw consistency, suggesting a goodness of fit. These two configurations were labelled ‘1’, while the rest were labelled ‘0’ (see Table 5). Both standard and specific analyses were run, following different, complementary guidelines [26,38].

**Table 5.** Truth table with presence and absence of outcome (Reduction in PHQ9 scores), minimised and sorted

Counselling uptake	Group supervision	Stigma	Quality of clinic management	Additional counselling	APC training	CCS training	Counsellor fidelity	Referrals	Number	PHQ9	Raw consist.	PRI consist.	SYM consist
*Presence of Reduction in PHQ09 scores*													
0	0	1	0	0	0	0	1	1	1	1	1.000000	1.000000	1.000000
1	1	1	0	1	1	0	1	0	1	1	0.858824	0.858824	0.858824
1	1	1	1	0	1	0	0	1	1	0	0.506494	0.000000	0.000000
1	0	1	1	1	0	1	1	0	1	0	0.061538	0.000000	0.000000
1	1	1	1	0	0	0	1	0	1	0	0.041667	0.000000	0.000000
1	1	1	1	1	1	0	1	1	1	0	0.037500	0.000000	0.000000
0	0	1	1	0	1	0	1	1	1	0	0.033898	0.000000	0.000000
1	1	1	0	0	1	0	0	0	1	0	0.000000	0.000000	0.000000
*Absence of Reduction in PHQ09 scores*													
0	0	1	1	0	1	0	1	1	1	1	1.000000	1.000000	1.000000
1	0	1	1	1	0	1	1	0	1	1	1.000000	1.000000	1.000000
1	1	1	0	0	1	0	0	0	1	1	1.000000	1.000000	1.000000
1	1	1	1	0	0	0	1	0	1	1	1.000000	1.000000	1.000000
1	1	1	1	1	1	0	1	1	1	1	1.000000	1.000000	1.000000
1	1	1	1	0	1	0	0	1	1	1	0.857143	0.710526	1.000000
0	0	1	0	0	0	0	1	1	1	0	0.507692	0.000000	0.000000
1	1	1	0	1	1	0	1	0	1	0	0.141176	0.141176	0.141176

First, a Standard Analysis was run, which is a first round of minimisation for which counterfactuals were identified, specifying key conditions for the simplification process. This function in fsQCA software provides a standard minimisation, emphasising the utility of an intermediate solution. In this analysis. It was assumed that all conditions should be theoretically present for a Reduction in PHQ9 scores to occur, apart from seeking additional counselling, the Ideal Clinic status of the clinic, and Stigma, which were assumed tobe either present or absent. The results of these steps are presented below in Table 6.

**Table 6.** Results of the standard sufficiency analysis

Solutions					
Outcome: PHQ9					
Complex			Raw coverage	Unique coverage	Consistency
~Counselling uptake*~Group supervision*Stigma*~Quality of clinic management*~Additional counselling*~APC training*~CCS training * Counsellor fidelity*Referrals			0.242537	0.242537	1.000000
Counselling uptake*Group supervision*Stigma*~Quality of clinic management*Additional counselling*APC training*~CCS training* Counsellor fidelity*~Referrals			0.272388	0.272388	0.858824
solution coverage: 0.514925	solution consistency: 0.920000				
Intermediate			Raw coverage	Unique coverage	Consistency
Referrals*Counsellor fidelity*~Additional counselling*~Quality of clinic management*Stigma			0.317164	0.250000	0.674603
Counsellor fidelity*APC training*Additional counselling*~Quality of clinic management*Stigma*Group supervision*Counselling uptake			0.294776	0.227612	0.868132
solution coverage: 0.544776		solution consistency: 0.780749			
Parsimonious			Raw coverage	Unique coverage	Consistency
~Quality of clinic management*Counsellor fidelity			0.623134	0.623134	0.755656
solution coverage: 0.623134		solution consistency: 0.755656			
Outcome: ~PHQ9					
Complex			Raw coverage	Unique coverage	Consistency
Counselling uptake*Group supervision*Stigma*~Quality of clinic management*~Additional counselling*APC training*~CCS training*~Counsellor fidelity*~Referrals			0.096519	0.096519	1.000000
Counselling uptake*Group supervision*Stigma*Quality of clinic management*~Additional counselling*~APC training*~CCS training*Counsellor fidelity*~Referrals			0.113924	0.099684	1.000000
~Counselling uptake*~Group supervision*Stigma*Quality of clinic management*~Additional counselling*APC training*~CCS training*Counsellor fidelity*Referrals			0.093354	0.079114	1.000000
Counselling uptake*~Group supervision*Stigma*Quality of clinic management*Additional counselling*~APC training*CCS training*Counsellor fidelity*~Referrals			0.102848	0.088608	1.000000
Counselling uptake*Group supervision*Stigma*Quality of clinic management*~Additional counselling*APC training*~CCS training*~Counsellor fidelity*Referrals			0.104430	0.094937	0.857143
Counselling uptake*Group supervision*Stigma*Quality of clinic management*Additional counselling*APC training*~CCS training*Counsellor fidelity*Referrals			0.126582	0.112342	1.000000
solution coverage: 0.599684		solution consistency: 0.971795			
Intermediate			Raw coverage	Unique coverage	Consistency
~Counsellor fidelity*~CCS training*Quality of clinic management*Stigma			0.183544	0.055380	0.906250
~CCS training*Additional counselling*Quality of clinic management*Stigma			0.295886	0.112342	0.869767
~CCS training*Quality of clinic management*Stigma*~Group supervision*~Counselling uptake			0.153481	0.064873	0.776000
~Referrals*~CCS training*~APC training*Quality of clinic management*Stigma			0.257911	0.099684	0.936782
~Referrals*~Counsellor fidelity*~CCS training*APC training*~Additional counselling*Stigma			0.162975	0.000000	1.000000
~Referrals*~Counsellor fidelity*~CCS training*~Additional counselling*Stigma*Group supervision			0.145570	0.000000	1.000000
~Referrals*~Counsellor fidelity*~CCS training*~Additional counselling*Stigma*Counselling uptake			0.162975	0.000000	1.000000
~Referrals*~APC training*Additional counselling*Quality of clinic management*Stigma*~Group supervision			0.175633	0.044304	0.909836
solution coverage: 0.680380		solution consistency: 0.914894			
Parsimonious			Raw coverage	Unique coverage	Consistency
Quality of clinic management			0.789557	0.375000	0.831667
Counselling uptake*~Additional counselling			0.504747	0.000000	0.914040
Group supervision*~Additional counselling			0.430380	0.000000	0.900662
~Additional counselling*APC training			0.506329	0.039557	0.909091
solution coverage: 0.981013		solution consistency: 0.836707			

As suggested in Table 6, the complex and intermediate solutions for a Reduction in PHQ9 scores were too broad to be helpful, and more minimisation was warranted. Further, the raw coverage scores for the solutions presented were far too low to robustly support any claims. The parsimonious solution seemed to be the most promising. In this ‘recipe’, the absence of Ideal Clinic scores (Quality of clinic management condition) combined with the presence of Counselling fidelity was sufficient for PHQ9 reduction with a consistency score of 0.755656 (while not especially high, still substantially higher than other combinations), and raw and unique coverage scores higher than 0.5.

Following this step, a Specific Analysis was run on the truth table, a function in fsQCA software that allows for manual selection of minimisation options, based on logical, intuitive expressions of causal pathways, which results in the generation of a most and least parsimonious solution (Table 7). These solutions suggested that the presence of all 10 conditions is sufficient to ensure a reduction in depression scores. However, it suggests that counselling uptake, group supervision, organisation, other forms of counselling, APC training and CCS training scores make little difference, and can be reduced to stigma, Counselling fidelity and Referrals. When applying the most parsimonious fsQCA algorithm, Counselling fidelity remains as the most sufficient condition for Reduction in PHQ9 scores, with a consistency of 0.76 (its combination with ~Quality of clinic management can be reduced to Counselling fidelity alone, given the absence of Quality of clinic management as a condition in the solution). Our findings therefore suggest that none of the conditions were necessary for a Reduction in PHQ9 scores to occur, while Counselling fidelity to the programme was a sufficient condition for this to occur.

**Table 7.** Results of the specific sufficiency analysis

Least parsimonious		Raw coverage	Unique coverage	Consistency
~Counselling uptake*~Group supervision*stigma*~Quality of clinic management*~Additional counselling*~APC training*~CCS training*Counsellor fidelity*Referrals		0.242537	0.242537	1.000000
Counselling uptake*Group supervision*Stigma*~Quality of clinic management*Additional counselling*APC training*~CCS training*Counsellor fidelity*~Referrals		0.272388	0.272388	0.858824
solution coverage: 0.514925	solution consistency: 0.920000			
Most parsimonious		Raw coverage	Unique coverage	Consistency
~Quality of clinic management*Counsellor fidelity		0.623134	0.623134	0.755656
solution coverage: 0.623134	solution consistency: 0.755656			

## Discussion

The principal purpose of this paper was to explore factors that could have contributed to a lower reduction in depression scores in the PRIME mental health integration project in North West province, South Africa. Investigating causal pathways as well as the outcomes of complex trials is a well-established goal in understanding social phenomena [36,39–41], and fsQCA – along with selected qualitative insights – provide good potential in demystifying change elements [25–27,31,39]. In this study, we theorised that a reduction in depression scores among the study population would be influenced by several possible causal pathways (see Figure 1). A particularly useful feature of QCA is its potential to test theory, in its ability to identify different conditions that are necessary and sufficient for an outcome to occur [42].

The key finding from this analysis was that, for the PRIME intervention in the North West province, fidelity of the counsellors to the programme was the most sufficient condition that predicted a reduction of PHQ9 scores among the target population. In this analysis, Counselling fidelity was a qualitative indication of counsellor adherence to the PRIME components as well as the dose of training and counselling they participated in. Counselling fidelity is a well-known moderator in the relationship between intended implementation and the outcomes of interventions [43–46]. Programme fidelity is multifaceted, and includes dimensions such as adherence, dose, participant responsiveness, quality of delivery, and programme differentiation [47]. The content of the intervention can be conceptualised as ‘active ingredients’ for the outcome to occur, and adherence by the programme stakeholders to the intervention – the frequency, duration, coverage of intervention elements – is a key determinant of the degree to which researchers can achieve the outcomes as planned [43]. However, the relationship between fidelity of the PRIME counsellors to the intervention components and an ultimate Reduction in PHQ9 scores is far from clear. This non-linear relationship, while suggested to be important, will no doubt be moderated and modified by several factors, such as the complexity of the intervention, external environmental factors, and personal characteristics of the stakeholders [48].

Accordingly, the other conditions considered in this analysis might still hold a fair degree of importance. A possibly important implementation condition that could have affected the trial outcome was the rate of referrals for counselling. It was previously reported that, while 1 400 referrals were made to lay counsellors during the trial period, only 11.3% of trial intervention group participants were referred to lay counsellors, and only 5% were referred to mental health specialists – this represents relatively low rates of referral, which could have had an influence on other conditions in the analysis [12]. A well-functioning referral structure is critical to achieve a continuum of care on PHC level [49], and nurses’ referral rates have been suggested to be higher in cases where pathways were set within the healthcare facility, where referral processes are explicit and well-described [50]. This being said, it is well-known that various structural health system limitations negatively affect mental health referrals from frontline PHC health workers to specialist care [51]. The PRIME intervention addressed this gap by introducing lay counsellors into the PHC clinic sphere, where they can collaborate with frontline nurses to provide support for depressive symptoms. It is well-established that collaborative care on the PHC level leads to positive outcomes, including significant reductions in depression symptoms over the short and medium term, improvements of mental health quality of life, social functioning and service satisfaction [52,53]. Further, counsellors based in PHC settings can greatly reduce mental health service bottlenecks at specialist levels, as well as a lower burden on general practitioners and lower overall health service utilisation [54]. Given common challenges in fostering interprofessional collaboration in PHC [55–59], improved clinical communication skills can greatly enhance the quality of interactions between nurses and counsellors and smooth over the referral process. Clinical communication skills can improve the understanding of professional roles and responsibilities of nurses and enhance communication skills within the PHC setting, crucial elements in developing patient-centred collaborative care [60]. This can lead to improved quality through improved decision-making, by sensitising nurses to the knowledge and skills of counsellors [61]. The fact that CCS and APC training were not uniformly attended by nurses across the trial clinics could further contribute to low referrals, and may in turn be attributed to a relatively low priority given to mental health in PHC training contexts in South Africa [12].

It is important to note that fsQCA, while promising and useful, does not guarantee an ultimately ‘true’ reflection of reality, as causality in social research is a far more complex matter. This approach strives to generate a parsimonious explanation of specific phenomena, within an appropriate degree of consideration of complexity in the causal process [62]. This multiple conjectural approach to causation focuses on a combination of conditions that is perceived to be sufficient for the outcome to occur, however, additional contextual factors could moderate this relationship. For instance, the relative low levels of mental health literacy (and associated low levels of health seeking) among patients were conjectured to possibly have contributed to low trial exposure, as well as the continuing real-life activities that render health systems complex – exemplified by the reported increased activity of specialist services provided by the district to control clinics, which could have further influenced the trial outcome [12].

This study has limitations. Incompatibilities in the data collected during the trial resulted in many variables being excluded in the final fsQCA analysis, thereby undercutting a full consideration of the possible configurational influences on the PRIME outcomes. The results presented are context-specific, as in any public health intervention, and generalisation to other settings should be interpreted with caution.

Yet, the findings from this fsQCA provide those working in strengthening public mental health systems with a formulation of ‘modest generalisation’, meaning that the importance of fidelity to a counselling intervention programme within a broader effort of integrating the identification and care of depressive symptoms into a PHC system could, with an appropriate degree of caution, be applied to similar cases with a reasonable range of similar characteristics [62]. While more modest than statistical inference, this generalisation to specific contexts is an important step towards strengthening the integration of mental healthcare into PHC in LMICs.

The utility of fsQCA as an evaluation tool is increasingly acknowledged, especially in its ability to reduce several data and measurement types into manageable comparisons [25]. The method is still very young compared to other comparative methods, and is constantly undergoing revisions and improvements in its assumptions and algorithms [63–65]. Nonetheless, even at this premature phase of its development as a recognised, robust evaluation methodology, there is clear promise for its use to ‘unravel the effects of a mental health interventions’ in LMICs [31]. However, this remains one step in a much longer process, and the application of iterative, responsive and robust implementation science approaches to scale up complex interventions such as PRIME is critical.

## Conclusion

There is ample evidence of the effectiveness of task-sharing as a strategy for integrating mental health care into primary health care in controlled settings [66]. There is, however, a paucity of evidence of how to employ this strategy successfully in real-world settings [67]. Findings from this evaluation of process data collected alongside the pragmatic PRIME trial in South Africa provides valuable insights into the finer implementation mechanisms involved in reducing depression scores and improving referral to task sharing counselling services within real world contexts. Nurses and lay counsellors have a palpable and critical role to play in primary mental health integration, and our study highlight the salience of programme fidelity in achieving this goal.

## Supplementary Material

Supplemental Material

## Data Availability

The raw data used during the current study are available from the corresponding author on reasonable request. Additional data from PRIME can be accessed on request from www.prime.uct.ac.za in line with PRIME’s data sharing policies.

## Paper context

Integrated primary mental healthcare is an established strategy to improve mental health systems. The PRIME intervention was an important step towards operationalising integration in the South African context. Our findings add valuable insights into the key mechanisms that influence integrated mental healthcare outcomes, specifically depression and referral rates. This paper highlights specific kinds of training and support required by nurses and lay counsellors to strengthen task-sharing and improving integration.
