# Supplementary material for: Fuzzy-set qualitative comparative analysis of implementation outcomes in an integrated mental healthcare trial in South Africa
Source: Glob Health Action. 2021 Aug 17;14(1):1940761. doi: 10.1080/16549716.2021.1940761 (PMC8381905; doi:10.1080/16549716.2021.1940761)
Supplement: Supplemental Material [file ZGHA_A_1940761_SM8162.docx]

ADDITIONAL FILE

| Facility name | Counselling fidelity | | Counselling uptake | | Group supervision coverage | | Stigma | | Referrals | |
| --- | --- | --- | --- | --- | --- | --- | --- | --- | --- | --- |
|  | Raw score | *Calibrated score* | Raw score | *Calibrated score* | Raw score | *Calibrated score* | Raw score | *Calibrated score* | Raw score | *Calibrated score* |
| Clinic A | 0,46 | *0,02* | 0,55 | *0,97* | 1 | *N/A* | 0,74 | *N/A* | 0,05 | *0,73* |
| Clinic B | 0,67 | *0,82* | 0,05 | *0,08* | 0 | *N/A* | 0,81 | *N/A* | 0,03 | *0,95* |
| Clinic C | 0,62 | *0,39* | 0,35 | *0,77* | 1 | *N/A* | 0,67 | *N/A* | 0,03 | *0,27* |
| Clinic D | 0,81 | *1* | 0,36 | *0,79* | 1 | *N/A* | 0,84 | *N/A* | 0,02 | *0,27* |
| Clinic E | 0,72 | *0,98* | 0,47 | *0,93* | 1 | *N/A* | 0,9 | *N/A* | 0,04 | *0,95* |
| Clinic F | 0,34 | *0,88* | 0,24 | *0,47* | 0 | *N/A* | 0,83 | *N/A* | 0,04 | *0,95* |
| Clinic G | 0,72 | *0,98* | 0,42 | *0,88* | 1 | *N/A* | 0,79 | *N/A* | 0,02 | *0,05* |
| Clinic H | 0,65 | *0,62* | 0,18 | *0,3* | 0 | *N/A* | 0,67 | *N/A* | 0,02 | *0,05* |
| Clinic I | 0,33 | *1* | 0,5 | *0,95* | 0 | *N/A* | 0,69 | *N/A* | 0,03 | *0,05* |
|  |  |  |  |  |  |  |  |  |  |  |
| Facility name | **Quality of clinic management (Ideal Clinic indicator)** | | **Any kind of other counselling**  **received for depression** | | **APC mental health training coverage** | | **CCS training coverage** | | **50%<PHQ9** | |
|  | Raw score | *Calibrated score* | Raw score | *Calibrated score* | Raw score | *Calibrated score* | Raw score | *Calibrated score* | Raw score | *Calibrated score* |
| Clinic A | 1,00 | *N/A* | 41,2 | *0,26* | 0,75 | *0,5* | 0,58 | *0,12* | 0,6 | *0,38* |
| Clinic B | 0,00 | *N/A* | 9,4 | *0,01* | 0,5 | *0,05* | 0,1 | *0* | 0,65 | *0,67* |
| Clinic C | 0,00 | *N/A* | 33,3 | *0,12* | 1 | *0,95* | 0,62 | *0,18* | 0,32 | *0* |
| Clinic D | 0,00 | *N/A* | 62,5 | *0,82* | 0,9 | *0,86* | 0,5 | *0,05* | 0,98 | *1* |
| Clinic E | 1,00 | *N/A* | 59,4 | *0,76* | 0,65 | *0,24* | 0,61 | *0,17* | 0,37 | *0* |
| Clinic F | 1,00 | *N/A* | 21,1 | *0,03* | 0,6 | *0,15* | 0,5 | *0,05* | 0,29 | *0* |
| Clinic G | 1,00 | *N/A* | 16 | *0,02* | 0,5 | *0,05* | 0,26 | *0* | 0,45 | *0,01* |
| Clinic H | 1,00 | *N/A* | 88,2 | *0,99* | 0,55 | *0,09* | 0,45 | *0,03* | 0,64 | *0,61* |
| Clinic I | 1,00 | *N/A* | 66,7 | *0,88* | 0,4 | *0,02* | 0,78 | *0,59* | 0,41 | *0,01* |

Table 1: Breakdown of raw and calibrated scores

Table 2: Reasons for exclusion of original variables

| Variable | Reason for exclusion |
| --- | --- |
| Staff complement | No differences between clinics, all clinics had the appropriate number of staff. |
| Presence of BPsych students | No differences between clinics, all clinics had students at 6 months, none had BPsych students available to an appropriate degree. |
| Visits by district psychologist | No differences between clinics, only one clinic had regular visits at 6 and 12 months. |
| Disruption of clinic services (Protest action, Facility removals) | No differences between clinics, only two events recorded during the time of measurement. |
| Individual supervision coverage | Concerns about measurement accuracy, not entirely clear how it was measured – group supervision coverage was a better indication of supervision. |
| Job strain | Average measurements of job strain per clinic were too closely clustered together to have an impact on the model. |
| Availability of counsellors | Little to no differentiation between clinics |
| General health | No logical role in hypothesised causal pathway |

Table 3: Final calibrated scores of variables included in fsQCA

| Name | Counselling uptake | Group supervision | Stigma | Quality of clinic management | Additional counselling | APC training | CCS training | Counsellor fidelity | Referrals | Reduction in PHQ9 |
| --- | --- | --- | --- | --- | --- | --- | --- | --- | --- | --- |
| Clinic A | 0,97 | 1 | 0,74 | 1 | 0,26 | 0,92 | 0,12 | 0,02 | 0,73 | 0,38 |
| Clinic B | 0,08 | 0 | 0,81 | 0 | 0,01 | 0,35 | 0 | 0,82 | 0,95 | 0,67 |
| Clinic C | 0,77 | 1 | 0,67 | 0 | 0,12 | 1 | 0,18 | 0,39 | 0,27 | 0 |
| Clinic D | 0,79 | 1 | 0,84 | 0 | 0,82 | 0,99 | 0,05 | 1 | 0,27 | 1 |
| Clinic E | 0,93 | 1 | 0,9 | 1 | 0,76 | 0,77 | 0,17 | 0,98 | 0,95 | 0 |
| Clinic F | 0,47 | 0 | 0,83 | 1 | 0,03 | 0,65 | 0,05 | 0,88 | 0,95 | 0 |
| Clinic G | 0,88 | 1 | 0,79 | 1 | 0,02 | 0,35 | 0 | 0,98 | 0,05 | 0,01 |
| Clinic H | 0,3 | 0 | 0,67 | 1 | 0,99 | 0,5 | 0,03 | 0,62 | 0,05 | 0,61 |
| Clinic I | 0,95 | 0 | 0,69 | 1 | 0,88 | 0,14 | 0,59 | 1 | 0,05 | 0,01 |

Table 4: Raw truth table with presence of outcome (reduction in PHQ9 scores)

| uptake | groupsupervisio | stigma | management | othercounsellin | apc_cal | ccs | fidelity_cal | referrals | number | phq9 | raw consist. | PRI consist. | SYM consist |
| --- | --- | --- | --- | --- | --- | --- | --- | --- | --- | --- | --- | --- | --- |
| 1 | 1 | 1 | 1 | 1 | 1 | 0 | 1 | 1 | 1 |  | 0.037500 | 0.000000 | 0.000000 |
| 1 | 1 | 1 | 1 | 0 | 1 | 0 | 0 | 1 | 1 |  | 0.506494 | 0.000000 | 0.000000 |
| 1 | 1 | 1 | 1 | 0 | 0 | 0 | 1 | 0 | 1 |  | 0.041667 | 0.000000 | 0.000000 |
| 1 | 1 | 1 | 0 | 1 | 1 | 0 | 1 | 0 | 1 |  | 0.858824 | 0.858824 | 0.858824 |
| 1 | 1 | 1 | 0 | 0 | 1 | 0 | 0 | 0 | 1 |  | 0.000000 | 0.000000 | 0.000000 |
| 1 | 0 | 1 | 1 | 1 | 0 | 1 | 1 | 0 | 1 |  | 0.061538 | 0.000000 | 0.000000 |
| 0 | 0 | 1 | 1 | 0 | 1 | 0 | 1 | 1 | 1 |  | 0.033898 | 0.000000 | 0.000000 |
| 0 | 0 | 1 | 0 | 0 | 0 | 0 | 1 | 1 | 1 |  | 1.000000 | 1.000000 | 1.000000 |
| 1 | 1 | 1 | 1 | 1 | 1 | 1 | 1 | 1 | 0 |  |  |  |  |
| 1 | 1 | 1 | 1 | 1 | 1 | 1 | 1 | 0 | 0 |  |  |  |  |
| 1 | 1 | 1 | 1 | 1 | 1 | 1 | 0 | 1 | 0 |  |  |  |  |
| 1 | 1 | 1 | 1 | 1 | 1 | 1 | 0 | 0 | 0 |  |  |  |  |
| 1 | 1 | 1 | 1 | 1 | 1 | 0 | 1 | 0 | 0 |  |  |  |  |
| 1 | 1 | 1 | 1 | 1 | 1 | 0 | 0 | 1 | 0 |  |  |  |  |
| 1 | 1 | 1 | 1 | 1 | 1 | 0 | 0 | 0 | 0 |  |  |  |  |
| 1 | 1 | 1 | 1 | 1 | 0 | 1 | 1 | 1 | 0 |  |  |  |  |
| 1 | 1 | 1 | 1 | 1 | 0 | 1 | 1 | 0 | 0 |  |  |  |  |
| 1 | 1 | 1 | 1 | 1 | 0 | 1 | 0 | 1 | 0 |  |  |  |  |
| 1 | 1 | 1 | 1 | 1 | 0 | 1 | 0 | 0 | 0 |  |  |  |  |
| 1 | 1 | 1 | 1 | 1 | 0 | 0 | 1 | 1 | 0 |  |  |  |  |
| 1 | 1 | 1 | 1 | 1 | 0 | 0 | 1 | 0 | 0 |  |  |  |  |
| 1 | 1 | 1 | 1 | 1 | 0 | 0 | 0 | 1 | 0 |  |  |  |  |
| 1 | 1 | 1 | 1 | 1 | 0 | 0 | 0 | 0 | 0 |  |  |  |  |
| 1 | 1 | 1 | 1 | 0 | 1 | 1 | 1 | 1 | 0 |  |  |  |  |
| 1 | 1 | 1 | 1 | 0 | 1 | 1 | 1 | 0 | 0 |  |  |  |  |
| 1 | 1 | 1 | 1 | 0 | 1 | 1 | 0 | 1 | 0 |  |  |  |  |
| 1 | 1 | 1 | 1 | 0 | 1 | 1 | 0 | 0 | 0 |  |  |  |  |
| 1 | 1 | 1 | 1 | 0 | 1 | 0 | 1 | 1 | 0 |  |  |  |  |
| 1 | 1 | 1 | 1 | 0 | 1 | 0 | 1 | 0 | 0 |  |  |  |  |
| 1 | 1 | 1 | 1 | 0 | 1 | 0 | 0 | 0 | 0 |  |  |  |  |
| 1 | 1 | 1 | 1 | 0 | 0 | 1 | 1 | 1 | 0 |  |  |  |  |
| 1 | 1 | 1 | 1 | 0 | 0 | 1 | 1 | 0 | 0 |  |  |  |  |
| 1 | 1 | 1 | 1 | 0 | 0 | 1 | 0 | 1 | 0 |  |  |  |  |
| 1 | 1 | 1 | 1 | 0 | 0 | 1 | 0 | 0 | 0 |  |  |  |  |
| 1 | 1 | 1 | 1 | 0 | 0 | 0 | 1 | 1 | 0 |  |  |  |  |
| 1 | 1 | 1 | 1 | 0 | 0 | 0 | 0 | 1 | 0 |  |  |  |  |
| 1 | 1 | 1 | 1 | 0 | 0 | 0 | 0 | 0 | 0 |  |  |  |  |
| 1 | 1 | 1 | 0 | 1 | 1 | 1 | 1 | 1 | 0 |  |  |  |  |
| 1 | 1 | 1 | 0 | 1 | 1 | 1 | 1 | 0 | 0 |  |  |  |  |
| 1 | 1 | 1 | 0 | 1 | 1 | 1 | 0 | 1 | 0 |  |  |  |  |
| 1 | 1 | 1 | 0 | 1 | 1 | 1 | 0 | 0 | 0 |  |  |  |  |
| 1 | 1 | 1 | 0 | 1 | 1 | 0 | 1 | 1 | 0 |  |  |  |  |
| 1 | 1 | 1 | 0 | 1 | 1 | 0 | 0 | 1 | 0 |  |  |  |  |
| 1 | 1 | 1 | 0 | 1 | 1 | 0 | 0 | 0 | 0 |  |  |  |  |
| 1 | 1 | 1 | 0 | 1 | 0 | 1 | 1 | 1 | 0 |  |  |  |  |
| 1 | 1 | 1 | 0 | 1 | 0 | 1 | 1 | 0 | 0 |  |  |  |  |
| 1 | 1 | 1 | 0 | 1 | 0 | 1 | 0 | 1 | 0 |  |  |  |  |
| 1 | 1 | 1 | 0 | 1 | 0 | 1 | 0 | 0 | 0 |  |  |  |  |
| 1 | 1 | 1 | 0 | 1 | 0 | 0 | 1 | 1 | 0 |  |  |  |  |
| 1 | 1 | 1 | 0 | 1 | 0 | 0 | 1 | 0 | 0 |  |  |  |  |
| 1 | 1 | 1 | 0 | 1 | 0 | 0 | 0 | 1 | 0 |  |  |  |  |
| 1 | 1 | 1 | 0 | 1 | 0 | 0 | 0 | 0 | 0 |  |  |  |  |
| 1 | 1 | 1 | 0 | 0 | 1 | 1 | 1 | 1 | 0 |  |  |  |  |
| 1 | 1 | 1 | 0 | 0 | 1 | 1 | 1 | 0 | 0 |  |  |  |  |
| 1 | 1 | 1 | 0 | 0 | 1 | 1 | 0 | 1 | 0 |  |  |  |  |
| 1 | 1 | 1 | 0 | 0 | 1 | 1 | 0 | 0 | 0 |  |  |  |  |
| 1 | 1 | 1 | 0 | 0 | 1 | 0 | 1 | 1 | 0 |  |  |  |  |
| 1 | 1 | 1 | 0 | 0 | 1 | 0 | 1 | 0 | 0 |  |  |  |  |
| 1 | 1 | 1 | 0 | 0 | 1 | 0 | 0 | 1 | 0 |  |  |  |  |
| 1 | 1 | 1 | 0 | 0 | 0 | 1 | 1 | 1 | 0 |  |  |  |  |
| 1 | 1 | 1 | 0 | 0 | 0 | 1 | 1 | 0 | 0 |  |  |  |  |
| 1 | 1 | 1 | 0 | 0 | 0 | 1 | 0 | 1 | 0 |  |  |  |  |
| 1 | 1 | 1 | 0 | 0 | 0 | 1 | 0 | 0 | 0 |  |  |  |  |
| 1 | 1 | 1 | 0 | 0 | 0 | 0 | 1 | 1 | 0 |  |  |  |  |
| 1 | 1 | 1 | 0 | 0 | 0 | 0 | 1 | 0 | 0 |  |  |  |  |
| 1 | 1 | 1 | 0 | 0 | 0 | 0 | 0 | 1 | 0 |  |  |  |  |
| 1 | 1 | 1 | 0 | 0 | 0 | 0 | 0 | 0 | 0 |  |  |  |  |
| 1 | 1 | 0 | 1 | 1 | 1 | 1 | 1 | 1 | 0 |  |  |  |  |
| 1 | 1 | 0 | 1 | 1 | 1 | 1 | 1 | 0 | 0 |  |  |  |  |
| 1 | 1 | 0 | 1 | 1 | 1 | 1 | 0 | 1 | 0 |  |  |  |  |
| 1 | 1 | 0 | 1 | 1 | 1 | 1 | 0 | 0 | 0 |  |  |  |  |
| 1 | 1 | 0 | 1 | 1 | 1 | 0 | 1 | 1 | 0 |  |  |  |  |
| 1 | 1 | 0 | 1 | 1 | 1 | 0 | 1 | 0 | 0 |  |  |  |  |
| 1 | 1 | 0 | 1 | 1 | 1 | 0 | 0 | 1 | 0 |  |  |  |  |
| 1 | 1 | 0 | 1 | 1 | 1 | 0 | 0 | 0 | 0 |  |  |  |  |
| 1 | 1 | 0 | 1 | 1 | 0 | 1 | 1 | 1 | 0 |  |  |  |  |
| 1 | 1 | 0 | 1 | 1 | 0 | 1 | 1 | 0 | 0 |  |  |  |  |
| 1 | 1 | 0 | 1 | 1 | 0 | 1 | 0 | 1 | 0 |  |  |  |  |
| 1 | 1 | 0 | 1 | 1 | 0 | 1 | 0 | 0 | 0 |  |  |  |  |
| 1 | 1 | 0 | 1 | 1 | 0 | 0 | 1 | 1 | 0 |  |  |  |  |
| 1 | 1 | 0 | 1 | 1 | 0 | 0 | 1 | 0 | 0 |  |  |  |  |
| 1 | 1 | 0 | 1 | 1 | 0 | 0 | 0 | 1 | 0 |  |  |  |  |
| 1 | 1 | 0 | 1 | 1 | 0 | 0 | 0 | 0 | 0 |  |  |  |  |
| 1 | 1 | 0 | 1 | 0 | 1 | 1 | 1 | 1 | 0 |  |  |  |  |
| 1 | 1 | 0 | 1 | 0 | 1 | 1 | 1 | 0 | 0 |  |  |  |  |
| 1 | 1 | 0 | 1 | 0 | 1 | 1 | 0 | 1 | 0 |  |  |  |  |
| 1 | 1 | 0 | 1 | 0 | 1 | 1 | 0 | 0 | 0 |  |  |  |  |
| 1 | 1 | 0 | 1 | 0 | 1 | 0 | 1 | 1 | 0 |  |  |  |  |
| 1 | 1 | 0 | 1 | 0 | 1 | 0 | 1 | 0 | 0 |  |  |  |  |
| 1 | 1 | 0 | 1 | 0 | 1 | 0 | 0 | 1 | 0 |  |  |  |  |
| 1 | 1 | 0 | 1 | 0 | 1 | 0 | 0 | 0 | 0 |  |  |  |  |
| 1 | 1 | 0 | 1 | 0 | 0 | 1 | 1 | 1 | 0 |  |  |  |  |
| 1 | 1 | 0 | 1 | 0 | 0 | 1 | 1 | 0 | 0 |  |  |  |  |
| 1 | 1 | 0 | 1 | 0 | 0 | 1 | 0 | 1 | 0 |  |  |  |  |
| 1 | 1 | 0 | 1 | 0 | 0 | 1 | 0 | 0 | 0 |  |  |  |  |
| 1 | 1 | 0 | 1 | 0 | 0 | 0 | 1 | 1 | 0 |  |  |  |  |
| 1 | 1 | 0 | 1 | 0 | 0 | 0 | 1 | 0 | 0 |  |  |  |  |
| 1 | 1 | 0 | 1 | 0 | 0 | 0 | 0 | 1 | 0 |  |  |  |  |
| 1 | 1 | 0 | 1 | 0 | 0 | 0 | 0 | 0 | 0 |  |  |  |  |
| 1 | 1 | 0 | 0 | 1 | 1 | 1 | 1 | 1 | 0 |  |  |  |  |
| 1 | 1 | 0 | 0 | 1 | 1 | 1 | 1 | 0 | 0 |  |  |  |  |
| 1 | 1 | 0 | 0 | 1 | 1 | 1 | 0 | 1 | 0 |  |  |  |  |
| 1 | 1 | 0 | 0 | 1 | 1 | 1 | 0 | 0 | 0 |  |  |  |  |
| 1 | 1 | 0 | 0 | 1 | 1 | 0 | 1 | 1 | 0 |  |  |  |  |
| 1 | 1 | 0 | 0 | 1 | 1 | 0 | 1 | 0 | 0 |  |  |  |  |
| 1 | 1 | 0 | 0 | 1 | 1 | 0 | 0 | 1 | 0 |  |  |  |  |
| 1 | 1 | 0 | 0 | 1 | 1 | 0 | 0 | 0 | 0 |  |  |  |  |
| 1 | 1 | 0 | 0 | 1 | 0 | 1 | 1 | 1 | 0 |  |  |  |  |
| 1 | 1 | 0 | 0 | 1 | 0 | 1 | 1 | 0 | 0 |  |  |  |  |
| 1 | 1 | 0 | 0 | 1 | 0 | 1 | 0 | 1 | 0 |  |  |  |  |
| 1 | 1 | 0 | 0 | 1 | 0 | 1 | 0 | 0 | 0 |  |  |  |  |
| 1 | 1 | 0 | 0 | 1 | 0 | 0 | 1 | 1 | 0 |  |  |  |  |
| 1 | 1 | 0 | 0 | 1 | 0 | 0 | 1 | 0 | 0 |  |  |  |  |
| 1 | 1 | 0 | 0 | 1 | 0 | 0 | 0 | 1 | 0 |  |  |  |  |
| 1 | 1 | 0 | 0 | 1 | 0 | 0 | 0 | 0 | 0 |  |  |  |  |
| 1 | 1 | 0 | 0 | 0 | 1 | 1 | 1 | 1 | 0 |  |  |  |  |
| 1 | 1 | 0 | 0 | 0 | 1 | 1 | 1 | 0 | 0 |  |  |  |  |
| 1 | 1 | 0 | 0 | 0 | 1 | 1 | 0 | 1 | 0 |  |  |  |  |
| 1 | 1 | 0 | 0 | 0 | 1 | 1 | 0 | 0 | 0 |  |  |  |  |
| 1 | 1 | 0 | 0 | 0 | 1 | 0 | 1 | 1 | 0 |  |  |  |  |
| 1 | 1 | 0 | 0 | 0 | 1 | 0 | 1 | 0 | 0 |  |  |  |  |
| 1 | 1 | 0 | 0 | 0 | 1 | 0 | 0 | 1 | 0 |  |  |  |  |
| 1 | 1 | 0 | 0 | 0 | 1 | 0 | 0 | 0 | 0 |  |  |  |  |
| 1 | 1 | 0 | 0 | 0 | 0 | 1 | 1 | 1 | 0 |  |  |  |  |
| 1 | 1 | 0 | 0 | 0 | 0 | 1 | 1 | 0 | 0 |  |  |  |  |
| 1 | 1 | 0 | 0 | 0 | 0 | 1 | 0 | 1 | 0 |  |  |  |  |
| 1 | 1 | 0 | 0 | 0 | 0 | 1 | 0 | 0 | 0 |  |  |  |  |
| 1 | 1 | 0 | 0 | 0 | 0 | 0 | 1 | 1 | 0 |  |  |  |  |
| 1 | 1 | 0 | 0 | 0 | 0 | 0 | 1 | 0 | 0 |  |  |  |  |
| 1 | 1 | 0 | 0 | 0 | 0 | 0 | 0 | 1 | 0 |  |  |  |  |
| 1 | 1 | 0 | 0 | 0 | 0 | 0 | 0 | 0 | 0 |  |  |  |  |
| 1 | 0 | 1 | 1 | 1 | 1 | 1 | 1 | 1 | 0 |  |  |  |  |
| 1 | 0 | 1 | 1 | 1 | 1 | 1 | 1 | 0 | 0 |  |  |  |  |
| 1 | 0 | 1 | 1 | 1 | 1 | 1 | 0 | 1 | 0 |  |  |  |  |
| 1 | 0 | 1 | 1 | 1 | 1 | 1 | 0 | 0 | 0 |  |  |  |  |
| 1 | 0 | 1 | 1 | 1 | 1 | 0 | 1 | 1 | 0 |  |  |  |  |
| 1 | 0 | 1 | 1 | 1 | 1 | 0 | 1 | 0 | 0 |  |  |  |  |
| 1 | 0 | 1 | 1 | 1 | 1 | 0 | 0 | 1 | 0 |  |  |  |  |
| 1 | 0 | 1 | 1 | 1 | 1 | 0 | 0 | 0 | 0 |  |  |  |  |
| 1 | 0 | 1 | 1 | 1 | 0 | 1 | 1 | 1 | 0 |  |  |  |  |
| 1 | 0 | 1 | 1 | 1 | 0 | 1 | 0 | 1 | 0 |  |  |  |  |
| 1 | 0 | 1 | 1 | 1 | 0 | 1 | 0 | 0 | 0 |  |  |  |  |
| 1 | 0 | 1 | 1 | 1 | 0 | 0 | 1 | 1 | 0 |  |  |  |  |
| 1 | 0 | 1 | 1 | 1 | 0 | 0 | 1 | 0 | 0 |  |  |  |  |
| 1 | 0 | 1 | 1 | 1 | 0 | 0 | 0 | 1 | 0 |  |  |  |  |
| 1 | 0 | 1 | 1 | 1 | 0 | 0 | 0 | 0 | 0 |  |  |  |  |
| 1 | 0 | 1 | 1 | 0 | 1 | 1 | 1 | 1 | 0 |  |  |  |  |
| 1 | 0 | 1 | 1 | 0 | 1 | 1 | 1 | 0 | 0 |  |  |  |  |
| 1 | 0 | 1 | 1 | 0 | 1 | 1 | 0 | 1 | 0 |  |  |  |  |
| 1 | 0 | 1 | 1 | 0 | 1 | 1 | 0 | 0 | 0 |  |  |  |  |
| 1 | 0 | 1 | 1 | 0 | 1 | 0 | 1 | 1 | 0 |  |  |  |  |
| 1 | 0 | 1 | 1 | 0 | 1 | 0 | 1 | 0 | 0 |  |  |  |  |
| 1 | 0 | 1 | 1 | 0 | 1 | 0 | 0 | 1 | 0 |  |  |  |  |
| 1 | 0 | 1 | 1 | 0 | 1 | 0 | 0 | 0 | 0 |  |  |  |  |
| 1 | 0 | 1 | 1 | 0 | 0 | 1 | 1 | 1 | 0 |  |  |  |  |
| 1 | 0 | 1 | 1 | 0 | 0 | 1 | 1 | 0 | 0 |  |  |  |  |
| 1 | 0 | 1 | 1 | 0 | 0 | 1 | 0 | 1 | 0 |  |  |  |  |
| 1 | 0 | 1 | 1 | 0 | 0 | 1 | 0 | 0 | 0 |  |  |  |  |
| 1 | 0 | 1 | 1 | 0 | 0 | 0 | 1 | 1 | 0 |  |  |  |  |
| 1 | 0 | 1 | 1 | 0 | 0 | 0 | 1 | 0 | 0 |  |  |  |  |
| 1 | 0 | 1 | 1 | 0 | 0 | 0 | 0 | 1 | 0 |  |  |  |  |
| 1 | 0 | 1 | 1 | 0 | 0 | 0 | 0 | 0 | 0 |  |  |  |  |
| 1 | 0 | 1 | 0 | 1 | 1 | 1 | 1 | 1 | 0 |  |  |  |  |
| 1 | 0 | 1 | 0 | 1 | 1 | 1 | 1 | 0 | 0 |  |  |  |  |
| 1 | 0 | 1 | 0 | 1 | 1 | 1 | 0 | 1 | 0 |  |  |  |  |
| 1 | 0 | 1 | 0 | 1 | 1 | 1 | 0 | 0 | 0 |  |  |  |  |
| 1 | 0 | 1 | 0 | 1 | 1 | 0 | 1 | 1 | 0 |  |  |  |  |
| 1 | 0 | 1 | 0 | 1 | 1 | 0 | 1 | 0 | 0 |  |  |  |  |
| 1 | 0 | 1 | 0 | 1 | 1 | 0 | 0 | 1 | 0 |  |  |  |  |
| 1 | 0 | 1 | 0 | 1 | 1 | 0 | 0 | 0 | 0 |  |  |  |  |
| 1 | 0 | 1 | 0 | 1 | 0 | 1 | 1 | 1 | 0 |  |  |  |  |
| 1 | 0 | 1 | 0 | 1 | 0 | 1 | 1 | 0 | 0 |  |  |  |  |
| 1 | 0 | 1 | 0 | 1 | 0 | 1 | 0 | 1 | 0 |  |  |  |  |
| 1 | 0 | 1 | 0 | 1 | 0 | 1 | 0 | 0 | 0 |  |  |  |  |
| 1 | 0 | 1 | 0 | 1 | 0 | 0 | 1 | 1 | 0 |  |  |  |  |
| 1 | 0 | 1 | 0 | 1 | 0 | 0 | 1 | 0 | 0 |  |  |  |  |
| 1 | 0 | 1 | 0 | 1 | 0 | 0 | 0 | 1 | 0 |  |  |  |  |
| 1 | 0 | 1 | 0 | 1 | 0 | 0 | 0 | 0 | 0 |  |  |  |  |
| 1 | 0 | 1 | 0 | 0 | 1 | 1 | 1 | 1 | 0 |  |  |  |  |
| 1 | 0 | 1 | 0 | 0 | 1 | 1 | 1 | 0 | 0 |  |  |  |  |
| 1 | 0 | 1 | 0 | 0 | 1 | 1 | 0 | 1 | 0 |  |  |  |  |
| 1 | 0 | 1 | 0 | 0 | 1 | 1 | 0 | 0 | 0 |  |  |  |  |
| 1 | 0 | 1 | 0 | 0 | 1 | 0 | 1 | 1 | 0 |  |  |  |  |
| 1 | 0 | 1 | 0 | 0 | 1 | 0 | 1 | 0 | 0 |  |  |  |  |
| 1 | 0 | 1 | 0 | 0 | 1 | 0 | 0 | 1 | 0 |  |  |  |  |
| 1 | 0 | 1 | 0 | 0 | 1 | 0 | 0 | 0 | 0 |  |  |  |  |
| 1 | 0 | 1 | 0 | 0 | 0 | 1 | 1 | 1 | 0 |  |  |  |  |
| 1 | 0 | 1 | 0 | 0 | 0 | 1 | 1 | 0 | 0 |  |  |  |  |
| 1 | 0 | 1 | 0 | 0 | 0 | 1 | 0 | 1 | 0 |  |  |  |  |
| 1 | 0 | 1 | 0 | 0 | 0 | 1 | 0 | 0 | 0 |  |  |  |  |
| 1 | 0 | 1 | 0 | 0 | 0 | 0 | 1 | 1 | 0 |  |  |  |  |
| 1 | 0 | 1 | 0 | 0 | 0 | 0 | 1 | 0 | 0 |  |  |  |  |
| 1 | 0 | 1 | 0 | 0 | 0 | 0 | 0 | 1 | 0 |  |  |  |  |
| 1 | 0 | 1 | 0 | 0 | 0 | 0 | 0 | 0 | 0 |  |  |  |  |
| 1 | 0 | 0 | 1 | 1 | 1 | 1 | 1 | 1 | 0 |  |  |  |  |
| 1 | 0 | 0 | 1 | 1 | 1 | 1 | 1 | 0 | 0 |  |  |  |  |
| 1 | 0 | 0 | 1 | 1 | 1 | 1 | 0 | 1 | 0 |  |  |  |  |
| 1 | 0 | 0 | 1 | 1 | 1 | 1 | 0 | 0 | 0 |  |  |  |  |
| 1 | 0 | 0 | 1 | 1 | 1 | 0 | 1 | 1 | 0 |  |  |  |  |
| 1 | 0 | 0 | 1 | 1 | 1 | 0 | 1 | 0 | 0 |  |  |  |  |
| 1 | 0 | 0 | 1 | 1 | 1 | 0 | 0 | 1 | 0 |  |  |  |  |
| 1 | 0 | 0 | 1 | 1 | 1 | 0 | 0 | 0 | 0 |  |  |  |  |
| 1 | 0 | 0 | 1 | 1 | 0 | 1 | 1 | 1 | 0 |  |  |  |  |
| 1 | 0 | 0 | 1 | 1 | 0 | 1 | 1 | 0 | 0 |  |  |  |  |
| 1 | 0 | 0 | 1 | 1 | 0 | 1 | 0 | 1 | 0 |  |  |  |  |
| 1 | 0 | 0 | 1 | 1 | 0 | 1 | 0 | 0 | 0 |  |  |  |  |
| 1 | 0 | 0 | 1 | 1 | 0 | 0 | 1 | 1 | 0 |  |  |  |  |
| 1 | 0 | 0 | 1 | 1 | 0 | 0 | 1 | 0 | 0 |  |  |  |  |
| 1 | 0 | 0 | 1 | 1 | 0 | 0 | 0 | 1 | 0 |  |  |  |  |
| 1 | 0 | 0 | 1 | 1 | 0 | 0 | 0 | 0 | 0 |  |  |  |  |
| 1 | 0 | 0 | 1 | 0 | 1 | 1 | 1 | 1 | 0 |  |  |  |  |
| 1 | 0 | 0 | 1 | 0 | 1 | 1 | 1 | 0 | 0 |  |  |  |  |
| 1 | 0 | 0 | 1 | 0 | 1 | 1 | 0 | 1 | 0 |  |  |  |  |
| 1 | 0 | 0 | 1 | 0 | 1 | 1 | 0 | 0 | 0 |  |  |  |  |
| 1 | 0 | 0 | 1 | 0 | 1 | 0 | 1 | 1 | 0 |  |  |  |  |
| 1 | 0 | 0 | 1 | 0 | 1 | 0 | 1 | 0 | 0 |  |  |  |  |
| 1 | 0 | 0 | 1 | 0 | 1 | 0 | 0 | 1 | 0 |  |  |  |  |
| 1 | 0 | 0 | 1 | 0 | 1 | 0 | 0 | 0 | 0 |  |  |  |  |
| 1 | 0 | 0 | 1 | 0 | 0 | 1 | 1 | 1 | 0 |  |  |  |  |
| 1 | 0 | 0 | 1 | 0 | 0 | 1 | 1 | 0 | 0 |  |  |  |  |
| 1 | 0 | 0 | 1 | 0 | 0 | 1 | 0 | 1 | 0 |  |  |  |  |
| 1 | 0 | 0 | 1 | 0 | 0 | 1 | 0 | 0 | 0 |  |  |  |  |
| 1 | 0 | 0 | 1 | 0 | 0 | 0 | 1 | 1 | 0 |  |  |  |  |
| 1 | 0 | 0 | 1 | 0 | 0 | 0 | 1 | 0 | 0 |  |  |  |  |
| 1 | 0 | 0 | 1 | 0 | 0 | 0 | 0 | 1 | 0 |  |  |  |  |
| 1 | 0 | 0 | 1 | 0 | 0 | 0 | 0 | 0 | 0 |  |  |  |  |
| 1 | 0 | 0 | 0 | 1 | 1 | 1 | 1 | 1 | 0 |  |  |  |  |
| 1 | 0 | 0 | 0 | 1 | 1 | 1 | 1 | 0 | 0 |  |  |  |  |
| 1 | 0 | 0 | 0 | 1 | 1 | 1 | 0 | 1 | 0 |  |  |  |  |
| 1 | 0 | 0 | 0 | 1 | 1 | 1 | 0 | 0 | 0 |  |  |  |  |
| 1 | 0 | 0 | 0 | 1 | 1 | 0 | 1 | 1 | 0 |  |  |  |  |
| 1 | 0 | 0 | 0 | 1 | 1 | 0 | 1 | 0 | 0 |  |  |  |  |
| 1 | 0 | 0 | 0 | 1 | 1 | 0 | 0 | 1 | 0 |  |  |  |  |
| 1 | 0 | 0 | 0 | 1 | 1 | 0 | 0 | 0 | 0 |  |  |  |  |
| 1 | 0 | 0 | 0 | 1 | 0 | 1 | 1 | 1 | 0 |  |  |  |  |
| 1 | 0 | 0 | 0 | 1 | 0 | 1 | 1 | 0 | 0 |  |  |  |  |
| 1 | 0 | 0 | 0 | 1 | 0 | 1 | 0 | 1 | 0 |  |  |  |  |
| 1 | 0 | 0 | 0 | 1 | 0 | 1 | 0 | 0 | 0 |  |  |  |  |
| 1 | 0 | 0 | 0 | 1 | 0 | 0 | 1 | 1 | 0 |  |  |  |  |
| 1 | 0 | 0 | 0 | 1 | 0 | 0 | 1 | 0 | 0 |  |  |  |  |
| 1 | 0 | 0 | 0 | 1 | 0 | 0 | 0 | 1 | 0 |  |  |  |  |
| 1 | 0 | 0 | 0 | 1 | 0 | 0 | 0 | 0 | 0 |  |  |  |  |
| 1 | 0 | 0 | 0 | 0 | 1 | 1 | 1 | 1 | 0 |  |  |  |  |
| 1 | 0 | 0 | 0 | 0 | 1 | 1 | 1 | 0 | 0 |  |  |  |  |
| 1 | 0 | 0 | 0 | 0 | 1 | 1 | 0 | 1 | 0 |  |  |  |  |
| 1 | 0 | 0 | 0 | 0 | 1 | 1 | 0 | 0 | 0 |  |  |  |  |
| 1 | 0 | 0 | 0 | 0 | 1 | 0 | 1 | 1 | 0 |  |  |  |  |
| 1 | 0 | 0 | 0 | 0 | 1 | 0 | 1 | 0 | 0 |  |  |  |  |
| 1 | 0 | 0 | 0 | 0 | 1 | 0 | 0 | 1 | 0 |  |  |  |  |
| 1 | 0 | 0 | 0 | 0 | 1 | 0 | 0 | 0 | 0 |  |  |  |  |
| 1 | 0 | 0 | 0 | 0 | 0 | 1 | 1 | 1 | 0 |  |  |  |  |
| 1 | 0 | 0 | 0 | 0 | 0 | 1 | 1 | 0 | 0 |  |  |  |  |
| 1 | 0 | 0 | 0 | 0 | 0 | 1 | 0 | 1 | 0 |  |  |  |  |
| 1 | 0 | 0 | 0 | 0 | 0 | 1 | 0 | 0 | 0 |  |  |  |  |
| 1 | 0 | 0 | 0 | 0 | 0 | 0 | 1 | 1 | 0 |  |  |  |  |
| 1 | 0 | 0 | 0 | 0 | 0 | 0 | 1 | 0 | 0 |  |  |  |  |
| 1 | 0 | 0 | 0 | 0 | 0 | 0 | 0 | 1 | 0 |  |  |  |  |
| 1 | 0 | 0 | 0 | 0 | 0 | 0 | 0 | 0 | 0 |  |  |  |  |
| 0 | 1 | 1 | 1 | 1 | 1 | 1 | 1 | 1 | 0 |  |  |  |  |
| 0 | 1 | 1 | 1 | 1 | 1 | 1 | 1 | 0 | 0 |  |  |  |  |
| 0 | 1 | 1 | 1 | 1 | 1 | 1 | 0 | 1 | 0 |  |  |  |  |
| 0 | 1 | 1 | 1 | 1 | 1 | 1 | 0 | 0 | 0 |  |  |  |  |
| 0 | 1 | 1 | 1 | 1 | 1 | 0 | 1 | 1 | 0 |  |  |  |  |
| 0 | 1 | 1 | 1 | 1 | 1 | 0 | 1 | 0 | 0 |  |  |  |  |
| 0 | 1 | 1 | 1 | 1 | 1 | 0 | 0 | 1 | 0 |  |  |  |  |
| 0 | 1 | 1 | 1 | 1 | 1 | 0 | 0 | 0 | 0 |  |  |  |  |
| 0 | 1 | 1 | 1 | 1 | 0 | 1 | 1 | 1 | 0 |  |  |  |  |
| 0 | 1 | 1 | 1 | 1 | 0 | 1 | 1 | 0 | 0 |  |  |  |  |
| 0 | 1 | 1 | 1 | 1 | 0 | 1 | 0 | 1 | 0 |  |  |  |  |
| 0 | 1 | 1 | 1 | 1 | 0 | 1 | 0 | 0 | 0 |  |  |  |  |
| 0 | 1 | 1 | 1 | 1 | 0 | 0 | 1 | 1 | 0 |  |  |  |  |
| 0 | 1 | 1 | 1 | 1 | 0 | 0 | 1 | 0 | 0 |  |  |  |  |
| 0 | 1 | 1 | 1 | 1 | 0 | 0 | 0 | 1 | 0 |  |  |  |  |
| 0 | 1 | 1 | 1 | 1 | 0 | 0 | 0 | 0 | 0 |  |  |  |  |
| 0 | 1 | 1 | 1 | 0 | 1 | 1 | 1 | 1 | 0 |  |  |  |  |
| 0 | 1 | 1 | 1 | 0 | 1 | 1 | 1 | 0 | 0 |  |  |  |  |
| 0 | 1 | 1 | 1 | 0 | 1 | 1 | 0 | 1 | 0 |  |  |  |  |
| 0 | 1 | 1 | 1 | 0 | 1 | 1 | 0 | 0 | 0 |  |  |  |  |
| 0 | 1 | 1 | 1 | 0 | 1 | 0 | 1 | 1 | 0 |  |  |  |  |
| 0 | 1 | 1 | 1 | 0 | 1 | 0 | 1 | 0 | 0 |  |  |  |  |
| 0 | 1 | 1 | 1 | 0 | 1 | 0 | 0 | 1 | 0 |  |  |  |  |
| 0 | 1 | 1 | 1 | 0 | 1 | 0 | 0 | 0 | 0 |  |  |  |  |
| 0 | 1 | 1 | 1 | 0 | 0 | 1 | 1 | 1 | 0 |  |  |  |  |
| 0 | 1 | 1 | 1 | 0 | 0 | 1 | 1 | 0 | 0 |  |  |  |  |
| 0 | 1 | 1 | 1 | 0 | 0 | 1 | 0 | 1 | 0 |  |  |  |  |
| 0 | 1 | 1 | 1 | 0 | 0 | 1 | 0 | 0 | 0 |  |  |  |  |
| 0 | 1 | 1 | 1 | 0 | 0 | 0 | 1 | 1 | 0 |  |  |  |  |
| 0 | 1 | 1 | 1 | 0 | 0 | 0 | 1 | 0 | 0 |  |  |  |  |
| 0 | 1 | 1 | 1 | 0 | 0 | 0 | 0 | 1 | 0 |  |  |  |  |
| 0 | 1 | 1 | 1 | 0 | 0 | 0 | 0 | 0 | 0 |  |  |  |  |
| 0 | 1 | 1 | 0 | 1 | 1 | 1 | 1 | 1 | 0 |  |  |  |  |
| 0 | 1 | 1 | 0 | 1 | 1 | 1 | 1 | 0 | 0 |  |  |  |  |
| 0 | 1 | 1 | 0 | 1 | 1 | 1 | 0 | 1 | 0 |  |  |  |  |
| 0 | 1 | 1 | 0 | 1 | 1 | 1 | 0 | 0 | 0 |  |  |  |  |
| 0 | 1 | 1 | 0 | 1 | 1 | 0 | 1 | 1 | 0 |  |  |  |  |
| 0 | 1 | 1 | 0 | 1 | 1 | 0 | 1 | 0 | 0 |  |  |  |  |
| 0 | 1 | 1 | 0 | 1 | 1 | 0 | 0 | 1 | 0 |  |  |  |  |
| 0 | 1 | 1 | 0 | 1 | 1 | 0 | 0 | 0 | 0 |  |  |  |  |
| 0 | 1 | 1 | 0 | 1 | 0 | 1 | 1 | 1 | 0 |  |  |  |  |
| 0 | 1 | 1 | 0 | 1 | 0 | 1 | 1 | 0 | 0 |  |  |  |  |
| 0 | 1 | 1 | 0 | 1 | 0 | 1 | 0 | 1 | 0 |  |  |  |  |
| 0 | 1 | 1 | 0 | 1 | 0 | 1 | 0 | 0 | 0 |  |  |  |  |
| 0 | 1 | 1 | 0 | 1 | 0 | 0 | 1 | 1 | 0 |  |  |  |  |
| 0 | 1 | 1 | 0 | 1 | 0 | 0 | 1 | 0 | 0 |  |  |  |  |
| 0 | 1 | 1 | 0 | 1 | 0 | 0 | 0 | 1 | 0 |  |  |  |  |
| 0 | 1 | 1 | 0 | 1 | 0 | 0 | 0 | 0 | 0 |  |  |  |  |
| 0 | 1 | 1 | 0 | 0 | 1 | 1 | 1 | 1 | 0 |  |  |  |  |
| 0 | 1 | 1 | 0 | 0 | 1 | 1 | 1 | 0 | 0 |  |  |  |  |
| 0 | 1 | 1 | 0 | 0 | 1 | 1 | 0 | 1 | 0 |  |  |  |  |
| 0 | 1 | 1 | 0 | 0 | 1 | 1 | 0 | 0 | 0 |  |  |  |  |
| 0 | 1 | 1 | 0 | 0 | 1 | 0 | 1 | 1 | 0 |  |  |  |  |
| 0 | 1 | 1 | 0 | 0 | 1 | 0 | 1 | 0 | 0 |  |  |  |  |
| 0 | 1 | 1 | 0 | 0 | 1 | 0 | 0 | 1 | 0 |  |  |  |  |
| 0 | 1 | 1 | 0 | 0 | 1 | 0 | 0 | 0 | 0 |  |  |  |  |
| 0 | 1 | 1 | 0 | 0 | 0 | 1 | 1 | 1 | 0 |  |  |  |  |
| 0 | 1 | 1 | 0 | 0 | 0 | 1 | 1 | 0 | 0 |  |  |  |  |
| 0 | 1 | 1 | 0 | 0 | 0 | 1 | 0 | 1 | 0 |  |  |  |  |
| 0 | 1 | 1 | 0 | 0 | 0 | 1 | 0 | 0 | 0 |  |  |  |  |
| 0 | 1 | 1 | 0 | 0 | 0 | 0 | 1 | 1 | 0 |  |  |  |  |
| 0 | 1 | 1 | 0 | 0 | 0 | 0 | 1 | 0 | 0 |  |  |  |  |
| 0 | 1 | 1 | 0 | 0 | 0 | 0 | 0 | 1 | 0 |  |  |  |  |
| 0 | 1 | 1 | 0 | 0 | 0 | 0 | 0 | 0 | 0 |  |  |  |  |
| 0 | 1 | 0 | 1 | 1 | 1 | 1 | 1 | 1 | 0 |  |  |  |  |
| 0 | 1 | 0 | 1 | 1 | 1 | 1 | 1 | 0 | 0 |  |  |  |  |
| 0 | 1 | 0 | 1 | 1 | 1 | 1 | 0 | 1 | 0 |  |  |  |  |
| 0 | 1 | 0 | 1 | 1 | 1 | 1 | 0 | 0 | 0 |  |  |  |  |
| 0 | 1 | 0 | 1 | 1 | 1 | 0 | 1 | 1 | 0 |  |  |  |  |
| 0 | 1 | 0 | 1 | 1 | 1 | 0 | 1 | 0 | 0 |  |  |  |  |
| 0 | 1 | 0 | 1 | 1 | 1 | 0 | 0 | 1 | 0 |  |  |  |  |
| 0 | 1 | 0 | 1 | 1 | 1 | 0 | 0 | 0 | 0 |  |  |  |  |
| 0 | 1 | 0 | 1 | 1 | 0 | 1 | 1 | 1 | 0 |  |  |  |  |
| 0 | 1 | 0 | 1 | 1 | 0 | 1 | 1 | 0 | 0 |  |  |  |  |
| 0 | 1 | 0 | 1 | 1 | 0 | 1 | 0 | 1 | 0 |  |  |  |  |
| 0 | 1 | 0 | 1 | 1 | 0 | 1 | 0 | 0 | 0 |  |  |  |  |
| 0 | 1 | 0 | 1 | 1 | 0 | 0 | 1 | 1 | 0 |  |  |  |  |
| 0 | 1 | 0 | 1 | 1 | 0 | 0 | 1 | 0 | 0 |  |  |  |  |
| 0 | 1 | 0 | 1 | 1 | 0 | 0 | 0 | 1 | 0 |  |  |  |  |
| 0 | 1 | 0 | 1 | 1 | 0 | 0 | 0 | 0 | 0 |  |  |  |  |
| 0 | 1 | 0 | 1 | 0 | 1 | 1 | 1 | 1 | 0 |  |  |  |  |
| 0 | 1 | 0 | 1 | 0 | 1 | 1 | 1 | 0 | 0 |  |  |  |  |
| 0 | 1 | 0 | 1 | 0 | 1 | 1 | 0 | 1 | 0 |  |  |  |  |
| 0 | 1 | 0 | 1 | 0 | 1 | 1 | 0 | 0 | 0 |  |  |  |  |
| 0 | 1 | 0 | 1 | 0 | 1 | 0 | 1 | 1 | 0 |  |  |  |  |
| 0 | 1 | 0 | 1 | 0 | 1 | 0 | 1 | 0 | 0 |  |  |  |  |
| 0 | 1 | 0 | 1 | 0 | 1 | 0 | 0 | 1 | 0 |  |  |  |  |
| 0 | 1 | 0 | 1 | 0 | 1 | 0 | 0 | 0 | 0 |  |  |  |  |
| 0 | 1 | 0 | 1 | 0 | 0 | 1 | 1 | 1 | 0 |  |  |  |  |
| 0 | 1 | 0 | 1 | 0 | 0 | 1 | 1 | 0 | 0 |  |  |  |  |
| 0 | 1 | 0 | 1 | 0 | 0 | 1 | 0 | 1 | 0 |  |  |  |  |
| 0 | 1 | 0 | 1 | 0 | 0 | 1 | 0 | 0 | 0 |  |  |  |  |
| 0 | 1 | 0 | 1 | 0 | 0 | 0 | 1 | 1 | 0 |  |  |  |  |
| 0 | 1 | 0 | 1 | 0 | 0 | 0 | 1 | 0 | 0 |  |  |  |  |
| 0 | 1 | 0 | 1 | 0 | 0 | 0 | 0 | 1 | 0 |  |  |  |  |
| 0 | 1 | 0 | 1 | 0 | 0 | 0 | 0 | 0 | 0 |  |  |  |  |
| 0 | 1 | 0 | 0 | 1 | 1 | 1 | 1 | 1 | 0 |  |  |  |  |
| 0 | 1 | 0 | 0 | 1 | 1 | 1 | 1 | 0 | 0 |  |  |  |  |
| 0 | 1 | 0 | 0 | 1 | 1 | 1 | 0 | 1 | 0 |  |  |  |  |
| 0 | 1 | 0 | 0 | 1 | 1 | 1 | 0 | 0 | 0 |  |  |  |  |
| 0 | 1 | 0 | 0 | 1 | 1 | 0 | 1 | 1 | 0 |  |  |  |  |
| 0 | 1 | 0 | 0 | 1 | 1 | 0 | 1 | 0 | 0 |  |  |  |  |
| 0 | 1 | 0 | 0 | 1 | 1 | 0 | 0 | 1 | 0 |  |  |  |  |
| 0 | 1 | 0 | 0 | 1 | 1 | 0 | 0 | 0 | 0 |  |  |  |  |
| 0 | 1 | 0 | 0 | 1 | 0 | 1 | 1 | 1 | 0 |  |  |  |  |
| 0 | 1 | 0 | 0 | 1 | 0 | 1 | 1 | 0 | 0 |  |  |  |  |
| 0 | 1 | 0 | 0 | 1 | 0 | 1 | 0 | 1 | 0 |  |  |  |  |
| 0 | 1 | 0 | 0 | 1 | 0 | 1 | 0 | 0 | 0 |  |  |  |  |
| 0 | 1 | 0 | 0 | 1 | 0 | 0 | 1 | 1 | 0 |  |  |  |  |
| 0 | 1 | 0 | 0 | 1 | 0 | 0 | 1 | 0 | 0 |  |  |  |  |
| 0 | 1 | 0 | 0 | 1 | 0 | 0 | 0 | 1 | 0 |  |  |  |  |
| 0 | 1 | 0 | 0 | 1 | 0 | 0 | 0 | 0 | 0 |  |  |  |  |
| 0 | 1 | 0 | 0 | 0 | 1 | 1 | 1 | 1 | 0 |  |  |  |  |
| 0 | 1 | 0 | 0 | 0 | 1 | 1 | 1 | 0 | 0 |  |  |  |  |
| 0 | 1 | 0 | 0 | 0 | 1 | 1 | 0 | 1 | 0 |  |  |  |  |
| 0 | 1 | 0 | 0 | 0 | 1 | 1 | 0 | 0 | 0 |  |  |  |  |
| 0 | 1 | 0 | 0 | 0 | 1 | 0 | 1 | 1 | 0 |  |  |  |  |
| 0 | 1 | 0 | 0 | 0 | 1 | 0 | 1 | 0 | 0 |  |  |  |  |
| 0 | 1 | 0 | 0 | 0 | 1 | 0 | 0 | 1 | 0 |  |  |  |  |
| 0 | 1 | 0 | 0 | 0 | 1 | 0 | 0 | 0 | 0 |  |  |  |  |
| 0 | 1 | 0 | 0 | 0 | 0 | 1 | 1 | 1 | 0 |  |  |  |  |
| 0 | 1 | 0 | 0 | 0 | 0 | 1 | 1 | 0 | 0 |  |  |  |  |
| 0 | 1 | 0 | 0 | 0 | 0 | 1 | 0 | 1 | 0 |  |  |  |  |
| 0 | 1 | 0 | 0 | 0 | 0 | 1 | 0 | 0 | 0 |  |  |  |  |
| 0 | 1 | 0 | 0 | 0 | 0 | 0 | 1 | 1 | 0 |  |  |  |  |
| 0 | 1 | 0 | 0 | 0 | 0 | 0 | 1 | 0 | 0 |  |  |  |  |
| 0 | 1 | 0 | 0 | 0 | 0 | 0 | 0 | 1 | 0 |  |  |  |  |
| 0 | 1 | 0 | 0 | 0 | 0 | 0 | 0 | 0 | 0 |  |  |  |  |
| 0 | 0 | 1 | 1 | 1 | 1 | 1 | 1 | 1 | 0 |  |  |  |  |
| 0 | 0 | 1 | 1 | 1 | 1 | 1 | 1 | 0 | 0 |  |  |  |  |
| 0 | 0 | 1 | 1 | 1 | 1 | 1 | 0 | 1 | 0 |  |  |  |  |
| 0 | 0 | 1 | 1 | 1 | 1 | 1 | 0 | 0 | 0 |  |  |  |  |
| 0 | 0 | 1 | 1 | 1 | 1 | 0 | 1 | 1 | 0 |  |  |  |  |
| 0 | 0 | 1 | 1 | 1 | 1 | 0 | 1 | 0 | 0 |  |  |  |  |
| 0 | 0 | 1 | 1 | 1 | 1 | 0 | 0 | 1 | 0 |  |  |  |  |
| 0 | 0 | 1 | 1 | 1 | 1 | 0 | 0 | 0 | 0 |  |  |  |  |
| 0 | 0 | 1 | 1 | 1 | 0 | 1 | 1 | 1 | 0 |  |  |  |  |
| 0 | 0 | 1 | 1 | 1 | 0 | 1 | 1 | 0 | 0 |  |  |  |  |
| 0 | 0 | 1 | 1 | 1 | 0 | 1 | 0 | 1 | 0 |  |  |  |  |
| 0 | 0 | 1 | 1 | 1 | 0 | 1 | 0 | 0 | 0 |  |  |  |  |
| 0 | 0 | 1 | 1 | 1 | 0 | 0 | 1 | 1 | 0 |  |  |  |  |
| 0 | 0 | 1 | 1 | 1 | 0 | 0 | 1 | 0 | 0 |  |  |  |  |
| 0 | 0 | 1 | 1 | 1 | 0 | 0 | 0 | 1 | 0 |  |  |  |  |
| 0 | 0 | 1 | 1 | 1 | 0 | 0 | 0 | 0 | 0 |  |  |  |  |
| 0 | 0 | 1 | 1 | 0 | 1 | 1 | 1 | 1 | 0 |  |  |  |  |
| 0 | 0 | 1 | 1 | 0 | 1 | 1 | 1 | 0 | 0 |  |  |  |  |
| 0 | 0 | 1 | 1 | 0 | 1 | 1 | 0 | 1 | 0 |  |  |  |  |
| 0 | 0 | 1 | 1 | 0 | 1 | 1 | 0 | 0 | 0 |  |  |  |  |
| 0 | 0 | 1 | 1 | 0 | 1 | 0 | 1 | 0 | 0 |  |  |  |  |
| 0 | 0 | 1 | 1 | 0 | 1 | 0 | 0 | 1 | 0 |  |  |  |  |
| 0 | 0 | 1 | 1 | 0 | 1 | 0 | 0 | 0 | 0 |  |  |  |  |
| 0 | 0 | 1 | 1 | 0 | 0 | 1 | 1 | 1 | 0 |  |  |  |  |
| 0 | 0 | 1 | 1 | 0 | 0 | 1 | 1 | 0 | 0 |  |  |  |  |
| 0 | 0 | 1 | 1 | 0 | 0 | 1 | 0 | 1 | 0 |  |  |  |  |
| 0 | 0 | 1 | 1 | 0 | 0 | 1 | 0 | 0 | 0 |  |  |  |  |
| 0 | 0 | 1 | 1 | 0 | 0 | 0 | 1 | 1 | 0 |  |  |  |  |
| 0 | 0 | 1 | 1 | 0 | 0 | 0 | 1 | 0 | 0 |  |  |  |  |
| 0 | 0 | 1 | 1 | 0 | 0 | 0 | 0 | 1 | 0 |  |  |  |  |
| 0 | 0 | 1 | 1 | 0 | 0 | 0 | 0 | 0 | 0 |  |  |  |  |
| 0 | 0 | 1 | 0 | 1 | 1 | 1 | 1 | 1 | 0 |  |  |  |  |
| 0 | 0 | 1 | 0 | 1 | 1 | 1 | 1 | 0 | 0 |  |  |  |  |
| 0 | 0 | 1 | 0 | 1 | 1 | 1 | 0 | 1 | 0 |  |  |  |  |
| 0 | 0 | 1 | 0 | 1 | 1 | 1 | 0 | 0 | 0 |  |  |  |  |
| 0 | 0 | 1 | 0 | 1 | 1 | 0 | 1 | 1 | 0 |  |  |  |  |
| 0 | 0 | 1 | 0 | 1 | 1 | 0 | 1 | 0 | 0 |  |  |  |  |
| 0 | 0 | 1 | 0 | 1 | 1 | 0 | 0 | 1 | 0 |  |  |  |  |
| 0 | 0 | 1 | 0 | 1 | 1 | 0 | 0 | 0 | 0 |  |  |  |  |
| 0 | 0 | 1 | 0 | 1 | 0 | 1 | 1 | 1 | 0 |  |  |  |  |
| 0 | 0 | 1 | 0 | 1 | 0 | 1 | 1 | 0 | 0 |  |  |  |  |
| 0 | 0 | 1 | 0 | 1 | 0 | 1 | 0 | 1 | 0 |  |  |  |  |
| 0 | 0 | 1 | 0 | 1 | 0 | 1 | 0 | 0 | 0 |  |  |  |  |
| 0 | 0 | 1 | 0 | 1 | 0 | 0 | 1 | 1 | 0 |  |  |  |  |
| 0 | 0 | 1 | 0 | 1 | 0 | 0 | 1 | 0 | 0 |  |  |  |  |
| 0 | 0 | 1 | 0 | 1 | 0 | 0 | 0 | 1 | 0 |  |  |  |  |
| 0 | 0 | 1 | 0 | 1 | 0 | 0 | 0 | 0 | 0 |  |  |  |  |
| 0 | 0 | 1 | 0 | 0 | 1 | 1 | 1 | 1 | 0 |  |  |  |  |
| 0 | 0 | 1 | 0 | 0 | 1 | 1 | 1 | 0 | 0 |  |  |  |  |
| 0 | 0 | 1 | 0 | 0 | 1 | 1 | 0 | 1 | 0 |  |  |  |  |
| 0 | 0 | 1 | 0 | 0 | 1 | 1 | 0 | 0 | 0 |  |  |  |  |
| 0 | 0 | 1 | 0 | 0 | 1 | 0 | 1 | 1 | 0 |  |  |  |  |
| 0 | 0 | 1 | 0 | 0 | 1 | 0 | 1 | 0 | 0 |  |  |  |  |
| 0 | 0 | 1 | 0 | 0 | 1 | 0 | 0 | 1 | 0 |  |  |  |  |
| 0 | 0 | 1 | 0 | 0 | 1 | 0 | 0 | 0 | 0 |  |  |  |  |
| 0 | 0 | 1 | 0 | 0 | 0 | 1 | 1 | 1 | 0 |  |  |  |  |
| 0 | 0 | 1 | 0 | 0 | 0 | 1 | 1 | 0 | 0 |  |  |  |  |
| 0 | 0 | 1 | 0 | 0 | 0 | 1 | 0 | 1 | 0 |  |  |  |  |
| 0 | 0 | 1 | 0 | 0 | 0 | 1 | 0 | 0 | 0 |  |  |  |  |
| 0 | 0 | 1 | 0 | 0 | 0 | 0 | 1 | 0 | 0 |  |  |  |  |
| 0 | 0 | 1 | 0 | 0 | 0 | 0 | 0 | 1 | 0 |  |  |  |  |
| 0 | 0 | 1 | 0 | 0 | 0 | 0 | 0 | 0 | 0 |  |  |  |  |
| 0 | 0 | 0 | 1 | 1 | 1 | 1 | 1 | 1 | 0 |  |  |  |  |
| 0 | 0 | 0 | 1 | 1 | 1 | 1 | 1 | 0 | 0 |  |  |  |  |
| 0 | 0 | 0 | 1 | 1 | 1 | 1 | 0 | 1 | 0 |  |  |  |  |
| 0 | 0 | 0 | 1 | 1 | 1 | 1 | 0 | 0 | 0 |  |  |  |  |
| 0 | 0 | 0 | 1 | 1 | 1 | 0 | 1 | 1 | 0 |  |  |  |  |
| 0 | 0 | 0 | 1 | 1 | 1 | 0 | 1 | 0 | 0 |  |  |  |  |
| 0 | 0 | 0 | 1 | 1 | 1 | 0 | 0 | 1 | 0 |  |  |  |  |
| 0 | 0 | 0 | 1 | 1 | 1 | 0 | 0 | 0 | 0 |  |  |  |  |
| 0 | 0 | 0 | 1 | 1 | 0 | 1 | 1 | 1 | 0 |  |  |  |  |
| 0 | 0 | 0 | 1 | 1 | 0 | 1 | 1 | 0 | 0 |  |  |  |  |
| 0 | 0 | 0 | 1 | 1 | 0 | 1 | 0 | 1 | 0 |  |  |  |  |
| 0 | 0 | 0 | 1 | 1 | 0 | 1 | 0 | 0 | 0 |  |  |  |  |
| 0 | 0 | 0 | 1 | 1 | 0 | 0 | 1 | 1 | 0 |  |  |  |  |
| 0 | 0 | 0 | 1 | 1 | 0 | 0 | 1 | 0 | 0 |  |  |  |  |
| 0 | 0 | 0 | 1 | 1 | 0 | 0 | 0 | 1 | 0 |  |  |  |  |
| 0 | 0 | 0 | 1 | 1 | 0 | 0 | 0 | 0 | 0 |  |  |  |  |
| 0 | 0 | 0 | 1 | 0 | 1 | 1 | 1 | 1 | 0 |  |  |  |  |
| 0 | 0 | 0 | 1 | 0 | 1 | 1 | 1 | 0 | 0 |  |  |  |  |
| 0 | 0 | 0 | 1 | 0 | 1 | 1 | 0 | 1 | 0 |  |  |  |  |
| 0 | 0 | 0 | 1 | 0 | 1 | 1 | 0 | 0 | 0 |  |  |  |  |
| 0 | 0 | 0 | 1 | 0 | 1 | 0 | 1 | 1 | 0 |  |  |  |  |
| 0 | 0 | 0 | 1 | 0 | 1 | 0 | 1 | 0 | 0 |  |  |  |  |
| 0 | 0 | 0 | 1 | 0 | 1 | 0 | 0 | 1 | 0 |  |  |  |  |
| 0 | 0 | 0 | 1 | 0 | 1 | 0 | 0 | 0 | 0 |  |  |  |  |
| 0 | 0 | 0 | 1 | 0 | 0 | 1 | 1 | 1 | 0 |  |  |  |  |
| 0 | 0 | 0 | 1 | 0 | 0 | 1 | 1 | 0 | 0 |  |  |  |  |
| 0 | 0 | 0 | 1 | 0 | 0 | 1 | 0 | 1 | 0 |  |  |  |  |
| 0 | 0 | 0 | 1 | 0 | 0 | 1 | 0 | 0 | 0 |  |  |  |  |
| 0 | 0 | 0 | 1 | 0 | 0 | 0 | 1 | 1 | 0 |  |  |  |  |
| 0 | 0 | 0 | 1 | 0 | 0 | 0 | 1 | 0 | 0 |  |  |  |  |
| 0 | 0 | 0 | 1 | 0 | 0 | 0 | 0 | 1 | 0 |  |  |  |  |
| 0 | 0 | 0 | 1 | 0 | 0 | 0 | 0 | 0 | 0 |  |  |  |  |
| 0 | 0 | 0 | 0 | 1 | 1 | 1 | 1 | 1 | 0 |  |  |  |  |
| 0 | 0 | 0 | 0 | 1 | 1 | 1 | 1 | 0 | 0 |  |  |  |  |
| 0 | 0 | 0 | 0 | 1 | 1 | 1 | 0 | 1 | 0 |  |  |  |  |
| 0 | 0 | 0 | 0 | 1 | 1 | 1 | 0 | 0 | 0 |  |  |  |  |
| 0 | 0 | 0 | 0 | 1 | 1 | 0 | 1 | 1 | 0 |  |  |  |  |
| 0 | 0 | 0 | 0 | 1 | 1 | 0 | 1 | 0 | 0 |  |  |  |  |
| 0 | 0 | 0 | 0 | 1 | 1 | 0 | 0 | 1 | 0 |  |  |  |  |
| 0 | 0 | 0 | 0 | 1 | 1 | 0 | 0 | 0 | 0 |  |  |  |  |
| 0 | 0 | 0 | 0 | 1 | 0 | 1 | 1 | 1 | 0 |  |  |  |  |
| 0 | 0 | 0 | 0 | 1 | 0 | 1 | 1 | 0 | 0 |  |  |  |  |
| 0 | 0 | 0 | 0 | 1 | 0 | 1 | 0 | 1 | 0 |  |  |  |  |
| 0 | 0 | 0 | 0 | 1 | 0 | 1 | 0 | 0 | 0 |  |  |  |  |
| 0 | 0 | 0 | 0 | 1 | 0 | 0 | 1 | 1 | 0 |  |  |  |  |
| 0 | 0 | 0 | 0 | 1 | 0 | 0 | 1 | 0 | 0 |  |  |  |  |
| 0 | 0 | 0 | 0 | 1 | 0 | 0 | 0 | 1 | 0 |  |  |  |  |
| 0 | 0 | 0 | 0 | 1 | 0 | 0 | 0 | 0 | 0 |  |  |  |  |
| 0 | 0 | 0 | 0 | 0 | 1 | 1 | 1 | 1 | 0 |  |  |  |  |
| 0 | 0 | 0 | 0 | 0 | 1 | 1 | 1 | 0 | 0 |  |  |  |  |
| 0 | 0 | 0 | 0 | 0 | 1 | 1 | 0 | 1 | 0 |  |  |  |  |
| 0 | 0 | 0 | 0 | 0 | 1 | 1 | 0 | 0 | 0 |  |  |  |  |
| 0 | 0 | 0 | 0 | 0 | 1 | 0 | 1 | 1 | 0 |  |  |  |  |
| 0 | 0 | 0 | 0 | 0 | 1 | 0 | 1 | 0 | 0 |  |  |  |  |
| 0 | 0 | 0 | 0 | 0 | 1 | 0 | 0 | 1 | 0 |  |  |  |  |
| 0 | 0 | 0 | 0 | 0 | 1 | 0 | 0 | 0 | 0 |  |  |  |  |
| 0 | 0 | 0 | 0 | 0 | 0 | 1 | 1 | 1 | 0 |  |  |  |  |
| 0 | 0 | 0 | 0 | 0 | 0 | 1 | 1 | 0 | 0 |  |  |  |  |
| 0 | 0 | 0 | 0 | 0 | 0 | 1 | 0 | 1 | 0 |  |  |  |  |
| 0 | 0 | 0 | 0 | 0 | 0 | 1 | 0 | 0 | 0 |  |  |  |  |
| 0 | 0 | 0 | 0 | 0 | 0 | 0 | 1 | 1 | 0 |  |  |  |  |
| 0 | 0 | 0 | 0 | 0 | 0 | 0 | 1 | 0 | 0 |  |  |  |  |
| 0 | 0 | 0 | 0 | 0 | 0 | 0 | 0 | 1 | 0 |  |  |  |  |
| 0 | 0 | 0 | 0 | 0 | 0 | 0 | 0 | 0 | 0 |  |  |  |  |

Table: Raw truth table with absence of outcome (reduction in PHQ9 scores)

| uptake | groupsupervisio | stigma | management | othercounsellin | apc_cal | ccs | fidelity_cal | referrals | number | ~phq9 | raw consist. | PRI consist. | SYM consist |
| --- | --- | --- | --- | --- | --- | --- | --- | --- | --- | --- | --- | --- | --- |
| 1 | 1 | 1 | 1 | 1 | 1 | 0 | 1 | 1 | 1 |  | 1.000000 | 1.000000 | 1.000000 |
| 1 | 1 | 1 | 1 | 0 | 1 | 0 | 0 | 1 | 1 |  | 0.857143 | 0.710526 | 1.000000 |
| 1 | 1 | 1 | 1 | 0 | 0 | 0 | 1 | 0 | 1 |  | 1.000000 | 1.000000 | 1.000000 |
| 1 | 1 | 1 | 0 | 1 | 1 | 0 | 1 | 0 | 1 |  | 0.141176 | 0.141176 | 0.141176 |
| 1 | 1 | 1 | 0 | 0 | 1 | 0 | 0 | 0 | 1 |  | 1.000000 | 1.000000 | 1.000000 |
| 1 | 0 | 1 | 1 | 1 | 0 | 1 | 1 | 0 | 1 |  | 1.000000 | 1.000000 | 1.000000 |
| 0 | 0 | 1 | 1 | 0 | 1 | 0 | 1 | 1 | 1 |  | 1.000000 | 1.000000 | 1.000000 |
| 0 | 0 | 1 | 0 | 0 | 0 | 0 | 1 | 1 | 1 |  | 0.507692 | 0.000000 | 0.000000 |
| 1 | 1 | 1 | 1 | 1 | 1 | 1 | 1 | 1 | 0 |  |  |  |  |
| 1 | 1 | 1 | 1 | 1 | 1 | 1 | 1 | 0 | 0 |  |  |  |  |
| 1 | 1 | 1 | 1 | 1 | 1 | 1 | 0 | 1 | 0 |  |  |  |  |
| 1 | 1 | 1 | 1 | 1 | 1 | 1 | 0 | 0 | 0 |  |  |  |  |
| 1 | 1 | 1 | 1 | 1 | 1 | 0 | 1 | 0 | 0 |  |  |  |  |
| 1 | 1 | 1 | 1 | 1 | 1 | 0 | 0 | 1 | 0 |  |  |  |  |
| 1 | 1 | 1 | 1 | 1 | 1 | 0 | 0 | 0 | 0 |  |  |  |  |
| 1 | 1 | 1 | 1 | 1 | 0 | 1 | 1 | 1 | 0 |  |  |  |  |
| 1 | 1 | 1 | 1 | 1 | 0 | 1 | 1 | 0 | 0 |  |  |  |  |
| 1 | 1 | 1 | 1 | 1 | 0 | 1 | 0 | 1 | 0 |  |  |  |  |
| 1 | 1 | 1 | 1 | 1 | 0 | 1 | 0 | 0 | 0 |  |  |  |  |
| 1 | 1 | 1 | 1 | 1 | 0 | 0 | 1 | 1 | 0 |  |  |  |  |
| 1 | 1 | 1 | 1 | 1 | 0 | 0 | 1 | 0 | 0 |  |  |  |  |
| 1 | 1 | 1 | 1 | 1 | 0 | 0 | 0 | 1 | 0 |  |  |  |  |
| 1 | 1 | 1 | 1 | 1 | 0 | 0 | 0 | 0 | 0 |  |  |  |  |
| 1 | 1 | 1 | 1 | 0 | 1 | 1 | 1 | 1 | 0 |  |  |  |  |
| 1 | 1 | 1 | 1 | 0 | 1 | 1 | 1 | 0 | 0 |  |  |  |  |
| 1 | 1 | 1 | 1 | 0 | 1 | 1 | 0 | 1 | 0 |  |  |  |  |
| 1 | 1 | 1 | 1 | 0 | 1 | 1 | 0 | 0 | 0 |  |  |  |  |
| 1 | 1 | 1 | 1 | 0 | 1 | 0 | 1 | 1 | 0 |  |  |  |  |
| 1 | 1 | 1 | 1 | 0 | 1 | 0 | 1 | 0 | 0 |  |  |  |  |
| 1 | 1 | 1 | 1 | 0 | 1 | 0 | 0 | 0 | 0 |  |  |  |  |
| 1 | 1 | 1 | 1 | 0 | 0 | 1 | 1 | 1 | 0 |  |  |  |  |
| 1 | 1 | 1 | 1 | 0 | 0 | 1 | 1 | 0 | 0 |  |  |  |  |
| 1 | 1 | 1 | 1 | 0 | 0 | 1 | 0 | 1 | 0 |  |  |  |  |
| 1 | 1 | 1 | 1 | 0 | 0 | 1 | 0 | 0 | 0 |  |  |  |  |
| 1 | 1 | 1 | 1 | 0 | 0 | 0 | 1 | 1 | 0 |  |  |  |  |
| 1 | 1 | 1 | 1 | 0 | 0 | 0 | 0 | 1 | 0 |  |  |  |  |
| 1 | 1 | 1 | 1 | 0 | 0 | 0 | 0 | 0 | 0 |  |  |  |  |
| 1 | 1 | 1 | 0 | 1 | 1 | 1 | 1 | 1 | 0 |  |  |  |  |
| 1 | 1 | 1 | 0 | 1 | 1 | 1 | 1 | 0 | 0 |  |  |  |  |
| 1 | 1 | 1 | 0 | 1 | 1 | 1 | 0 | 1 | 0 |  |  |  |  |
| 1 | 1 | 1 | 0 | 1 | 1 | 1 | 0 | 0 | 0 |  |  |  |  |
| 1 | 1 | 1 | 0 | 1 | 1 | 0 | 1 | 1 | 0 |  |  |  |  |
| 1 | 1 | 1 | 0 | 1 | 1 | 0 | 0 | 1 | 0 |  |  |  |  |
| 1 | 1 | 1 | 0 | 1 | 1 | 0 | 0 | 0 | 0 |  |  |  |  |
| 1 | 1 | 1 | 0 | 1 | 0 | 1 | 1 | 1 | 0 |  |  |  |  |
| 1 | 1 | 1 | 0 | 1 | 0 | 1 | 1 | 0 | 0 |  |  |  |  |
| 1 | 1 | 1 | 0 | 1 | 0 | 1 | 0 | 1 | 0 |  |  |  |  |
| 1 | 1 | 1 | 0 | 1 | 0 | 1 | 0 | 0 | 0 |  |  |  |  |
| 1 | 1 | 1 | 0 | 1 | 0 | 0 | 1 | 1 | 0 |  |  |  |  |
| 1 | 1 | 1 | 0 | 1 | 0 | 0 | 1 | 0 | 0 |  |  |  |  |
| 1 | 1 | 1 | 0 | 1 | 0 | 0 | 0 | 1 | 0 |  |  |  |  |
| 1 | 1 | 1 | 0 | 1 | 0 | 0 | 0 | 0 | 0 |  |  |  |  |
| 1 | 1 | 1 | 0 | 0 | 1 | 1 | 1 | 1 | 0 |  |  |  |  |
| 1 | 1 | 1 | 0 | 0 | 1 | 1 | 1 | 0 | 0 |  |  |  |  |
| 1 | 1 | 1 | 0 | 0 | 1 | 1 | 0 | 1 | 0 |  |  |  |  |
| 1 | 1 | 1 | 0 | 0 | 1 | 1 | 0 | 0 | 0 |  |  |  |  |
| 1 | 1 | 1 | 0 | 0 | 1 | 0 | 1 | 1 | 0 |  |  |  |  |
| 1 | 1 | 1 | 0 | 0 | 1 | 0 | 1 | 0 | 0 |  |  |  |  |
| 1 | 1 | 1 | 0 | 0 | 1 | 0 | 0 | 1 | 0 |  |  |  |  |
| 1 | 1 | 1 | 0 | 0 | 0 | 1 | 1 | 1 | 0 |  |  |  |  |
| 1 | 1 | 1 | 0 | 0 | 0 | 1 | 1 | 0 | 0 |  |  |  |  |
| 1 | 1 | 1 | 0 | 0 | 0 | 1 | 0 | 1 | 0 |  |  |  |  |
| 1 | 1 | 1 | 0 | 0 | 0 | 1 | 0 | 0 | 0 |  |  |  |  |
| 1 | 1 | 1 | 0 | 0 | 0 | 0 | 1 | 1 | 0 |  |  |  |  |
| 1 | 1 | 1 | 0 | 0 | 0 | 0 | 1 | 0 | 0 |  |  |  |  |
| 1 | 1 | 1 | 0 | 0 | 0 | 0 | 0 | 1 | 0 |  |  |  |  |
| 1 | 1 | 1 | 0 | 0 | 0 | 0 | 0 | 0 | 0 |  |  |  |  |
| 1 | 1 | 0 | 1 | 1 | 1 | 1 | 1 | 1 | 0 |  |  |  |  |
| 1 | 1 | 0 | 1 | 1 | 1 | 1 | 1 | 0 | 0 |  |  |  |  |
| 1 | 1 | 0 | 1 | 1 | 1 | 1 | 0 | 1 | 0 |  |  |  |  |
| 1 | 1 | 0 | 1 | 1 | 1 | 1 | 0 | 0 | 0 |  |  |  |  |
| 1 | 1 | 0 | 1 | 1 | 1 | 0 | 1 | 1 | 0 |  |  |  |  |
| 1 | 1 | 0 | 1 | 1 | 1 | 0 | 1 | 0 | 0 |  |  |  |  |
| 1 | 1 | 0 | 1 | 1 | 1 | 0 | 0 | 1 | 0 |  |  |  |  |
| 1 | 1 | 0 | 1 | 1 | 1 | 0 | 0 | 0 | 0 |  |  |  |  |
| 1 | 1 | 0 | 1 | 1 | 0 | 1 | 1 | 1 | 0 |  |  |  |  |
| 1 | 1 | 0 | 1 | 1 | 0 | 1 | 1 | 0 | 0 |  |  |  |  |
| 1 | 1 | 0 | 1 | 1 | 0 | 1 | 0 | 1 | 0 |  |  |  |  |
| 1 | 1 | 0 | 1 | 1 | 0 | 1 | 0 | 0 | 0 |  |  |  |  |
| 1 | 1 | 0 | 1 | 1 | 0 | 0 | 1 | 1 | 0 |  |  |  |  |
| 1 | 1 | 0 | 1 | 1 | 0 | 0 | 1 | 0 | 0 |  |  |  |  |
| 1 | 1 | 0 | 1 | 1 | 0 | 0 | 0 | 1 | 0 |  |  |  |  |
| 1 | 1 | 0 | 1 | 1 | 0 | 0 | 0 | 0 | 0 |  |  |  |  |
| 1 | 1 | 0 | 1 | 0 | 1 | 1 | 1 | 1 | 0 |  |  |  |  |
| 1 | 1 | 0 | 1 | 0 | 1 | 1 | 1 | 0 | 0 |  |  |  |  |
| 1 | 1 | 0 | 1 | 0 | 1 | 1 | 0 | 1 | 0 |  |  |  |  |
| 1 | 1 | 0 | 1 | 0 | 1 | 1 | 0 | 0 | 0 |  |  |  |  |
| 1 | 1 | 0 | 1 | 0 | 1 | 0 | 1 | 1 | 0 |  |  |  |  |
| 1 | 1 | 0 | 1 | 0 | 1 | 0 | 1 | 0 | 0 |  |  |  |  |
| 1 | 1 | 0 | 1 | 0 | 1 | 0 | 0 | 1 | 0 |  |  |  |  |
| 1 | 1 | 0 | 1 | 0 | 1 | 0 | 0 | 0 | 0 |  |  |  |  |
| 1 | 1 | 0 | 1 | 0 | 0 | 1 | 1 | 1 | 0 |  |  |  |  |
| 1 | 1 | 0 | 1 | 0 | 0 | 1 | 1 | 0 | 0 |  |  |  |  |
| 1 | 1 | 0 | 1 | 0 | 0 | 1 | 0 | 1 | 0 |  |  |  |  |
| 1 | 1 | 0 | 1 | 0 | 0 | 1 | 0 | 0 | 0 |  |  |  |  |
| 1 | 1 | 0 | 1 | 0 | 0 | 0 | 1 | 1 | 0 |  |  |  |  |
| 1 | 1 | 0 | 1 | 0 | 0 | 0 | 1 | 0 | 0 |  |  |  |  |
| 1 | 1 | 0 | 1 | 0 | 0 | 0 | 0 | 1 | 0 |  |  |  |  |
| 1 | 1 | 0 | 1 | 0 | 0 | 0 | 0 | 0 | 0 |  |  |  |  |
| 1 | 1 | 0 | 0 | 1 | 1 | 1 | 1 | 1 | 0 |  |  |  |  |
| 1 | 1 | 0 | 0 | 1 | 1 | 1 | 1 | 0 | 0 |  |  |  |  |
| 1 | 1 | 0 | 0 | 1 | 1 | 1 | 0 | 1 | 0 |  |  |  |  |
| 1 | 1 | 0 | 0 | 1 | 1 | 1 | 0 | 0 | 0 |  |  |  |  |
| 1 | 1 | 0 | 0 | 1 | 1 | 0 | 1 | 1 | 0 |  |  |  |  |
| 1 | 1 | 0 | 0 | 1 | 1 | 0 | 1 | 0 | 0 |  |  |  |  |
| 1 | 1 | 0 | 0 | 1 | 1 | 0 | 0 | 1 | 0 |  |  |  |  |
| 1 | 1 | 0 | 0 | 1 | 1 | 0 | 0 | 0 | 0 |  |  |  |  |
| 1 | 1 | 0 | 0 | 1 | 0 | 1 | 1 | 1 | 0 |  |  |  |  |
| 1 | 1 | 0 | 0 | 1 | 0 | 1 | 1 | 0 | 0 |  |  |  |  |
| 1 | 1 | 0 | 0 | 1 | 0 | 1 | 0 | 1 | 0 |  |  |  |  |
| 1 | 1 | 0 | 0 | 1 | 0 | 1 | 0 | 0 | 0 |  |  |  |  |
| 1 | 1 | 0 | 0 | 1 | 0 | 0 | 1 | 1 | 0 |  |  |  |  |
| 1 | 1 | 0 | 0 | 1 | 0 | 0 | 1 | 0 | 0 |  |  |  |  |
| 1 | 1 | 0 | 0 | 1 | 0 | 0 | 0 | 1 | 0 |  |  |  |  |
| 1 | 1 | 0 | 0 | 1 | 0 | 0 | 0 | 0 | 0 |  |  |  |  |
| 1 | 1 | 0 | 0 | 0 | 1 | 1 | 1 | 1 | 0 |  |  |  |  |
| 1 | 1 | 0 | 0 | 0 | 1 | 1 | 1 | 0 | 0 |  |  |  |  |
| 1 | 1 | 0 | 0 | 0 | 1 | 1 | 0 | 1 | 0 |  |  |  |  |
| 1 | 1 | 0 | 0 | 0 | 1 | 1 | 0 | 0 | 0 |  |  |  |  |
| 1 | 1 | 0 | 0 | 0 | 1 | 0 | 1 | 1 | 0 |  |  |  |  |
| 1 | 1 | 0 | 0 | 0 | 1 | 0 | 1 | 0 | 0 |  |  |  |  |
| 1 | 1 | 0 | 0 | 0 | 1 | 0 | 0 | 1 | 0 |  |  |  |  |
| 1 | 1 | 0 | 0 | 0 | 1 | 0 | 0 | 0 | 0 |  |  |  |  |
| 1 | 1 | 0 | 0 | 0 | 0 | 1 | 1 | 1 | 0 |  |  |  |  |
| 1 | 1 | 0 | 0 | 0 | 0 | 1 | 1 | 0 | 0 |  |  |  |  |
| 1 | 1 | 0 | 0 | 0 | 0 | 1 | 0 | 1 | 0 |  |  |  |  |
| 1 | 1 | 0 | 0 | 0 | 0 | 1 | 0 | 0 | 0 |  |  |  |  |
| 1 | 1 | 0 | 0 | 0 | 0 | 0 | 1 | 1 | 0 |  |  |  |  |
| 1 | 1 | 0 | 0 | 0 | 0 | 0 | 1 | 0 | 0 |  |  |  |  |
| 1 | 1 | 0 | 0 | 0 | 0 | 0 | 0 | 1 | 0 |  |  |  |  |
| 1 | 1 | 0 | 0 | 0 | 0 | 0 | 0 | 0 | 0 |  |  |  |  |
| 1 | 0 | 1 | 1 | 1 | 1 | 1 | 1 | 1 | 0 |  |  |  |  |
| 1 | 0 | 1 | 1 | 1 | 1 | 1 | 1 | 0 | 0 |  |  |  |  |
| 1 | 0 | 1 | 1 | 1 | 1 | 1 | 0 | 1 | 0 |  |  |  |  |
| 1 | 0 | 1 | 1 | 1 | 1 | 1 | 0 | 0 | 0 |  |  |  |  |
| 1 | 0 | 1 | 1 | 1 | 1 | 0 | 1 | 1 | 0 |  |  |  |  |
| 1 | 0 | 1 | 1 | 1 | 1 | 0 | 1 | 0 | 0 |  |  |  |  |
| 1 | 0 | 1 | 1 | 1 | 1 | 0 | 0 | 1 | 0 |  |  |  |  |
| 1 | 0 | 1 | 1 | 1 | 1 | 0 | 0 | 0 | 0 |  |  |  |  |
| 1 | 0 | 1 | 1 | 1 | 0 | 1 | 1 | 1 | 0 |  |  |  |  |
| 1 | 0 | 1 | 1 | 1 | 0 | 1 | 0 | 1 | 0 |  |  |  |  |
| 1 | 0 | 1 | 1 | 1 | 0 | 1 | 0 | 0 | 0 |  |  |  |  |
| 1 | 0 | 1 | 1 | 1 | 0 | 0 | 1 | 1 | 0 |  |  |  |  |
| 1 | 0 | 1 | 1 | 1 | 0 | 0 | 1 | 0 | 0 |  |  |  |  |
| 1 | 0 | 1 | 1 | 1 | 0 | 0 | 0 | 1 | 0 |  |  |  |  |
| 1 | 0 | 1 | 1 | 1 | 0 | 0 | 0 | 0 | 0 |  |  |  |  |
| 1 | 0 | 1 | 1 | 0 | 1 | 1 | 1 | 1 | 0 |  |  |  |  |
| 1 | 0 | 1 | 1 | 0 | 1 | 1 | 1 | 0 | 0 |  |  |  |  |
| 1 | 0 | 1 | 1 | 0 | 1 | 1 | 0 | 1 | 0 |  |  |  |  |
| 1 | 0 | 1 | 1 | 0 | 1 | 1 | 0 | 0 | 0 |  |  |  |  |
| 1 | 0 | 1 | 1 | 0 | 1 | 0 | 1 | 1 | 0 |  |  |  |  |
| 1 | 0 | 1 | 1 | 0 | 1 | 0 | 1 | 0 | 0 |  |  |  |  |
| 1 | 0 | 1 | 1 | 0 | 1 | 0 | 0 | 1 | 0 |  |  |  |  |
| 1 | 0 | 1 | 1 | 0 | 1 | 0 | 0 | 0 | 0 |  |  |  |  |
| 1 | 0 | 1 | 1 | 0 | 0 | 1 | 1 | 1 | 0 |  |  |  |  |
| 1 | 0 | 1 | 1 | 0 | 0 | 1 | 1 | 0 | 0 |  |  |  |  |
| 1 | 0 | 1 | 1 | 0 | 0 | 1 | 0 | 1 | 0 |  |  |  |  |
| 1 | 0 | 1 | 1 | 0 | 0 | 1 | 0 | 0 | 0 |  |  |  |  |
| 1 | 0 | 1 | 1 | 0 | 0 | 0 | 1 | 1 | 0 |  |  |  |  |
| 1 | 0 | 1 | 1 | 0 | 0 | 0 | 1 | 0 | 0 |  |  |  |  |
| 1 | 0 | 1 | 1 | 0 | 0 | 0 | 0 | 1 | 0 |  |  |  |  |
| 1 | 0 | 1 | 1 | 0 | 0 | 0 | 0 | 0 | 0 |  |  |  |  |
| 1 | 0 | 1 | 0 | 1 | 1 | 1 | 1 | 1 | 0 |  |  |  |  |
| 1 | 0 | 1 | 0 | 1 | 1 | 1 | 1 | 0 | 0 |  |  |  |  |
| 1 | 0 | 1 | 0 | 1 | 1 | 1 | 0 | 1 | 0 |  |  |  |  |
| 1 | 0 | 1 | 0 | 1 | 1 | 1 | 0 | 0 | 0 |  |  |  |  |
| 1 | 0 | 1 | 0 | 1 | 1 | 0 | 1 | 1 | 0 |  |  |  |  |
| 1 | 0 | 1 | 0 | 1 | 1 | 0 | 1 | 0 | 0 |  |  |  |  |
| 1 | 0 | 1 | 0 | 1 | 1 | 0 | 0 | 1 | 0 |  |  |  |  |
| 1 | 0 | 1 | 0 | 1 | 1 | 0 | 0 | 0 | 0 |  |  |  |  |
| 1 | 0 | 1 | 0 | 1 | 0 | 1 | 1 | 1 | 0 |  |  |  |  |
| 1 | 0 | 1 | 0 | 1 | 0 | 1 | 1 | 0 | 0 |  |  |  |  |
| 1 | 0 | 1 | 0 | 1 | 0 | 1 | 0 | 1 | 0 |  |  |  |  |
| 1 | 0 | 1 | 0 | 1 | 0 | 1 | 0 | 0 | 0 |  |  |  |  |
| 1 | 0 | 1 | 0 | 1 | 0 | 0 | 1 | 1 | 0 |  |  |  |  |
| 1 | 0 | 1 | 0 | 1 | 0 | 0 | 1 | 0 | 0 |  |  |  |  |
| 1 | 0 | 1 | 0 | 1 | 0 | 0 | 0 | 1 | 0 |  |  |  |  |
| 1 | 0 | 1 | 0 | 1 | 0 | 0 | 0 | 0 | 0 |  |  |  |  |
| 1 | 0 | 1 | 0 | 0 | 1 | 1 | 1 | 1 | 0 |  |  |  |  |
| 1 | 0 | 1 | 0 | 0 | 1 | 1 | 1 | 0 | 0 |  |  |  |  |
| 1 | 0 | 1 | 0 | 0 | 1 | 1 | 0 | 1 | 0 |  |  |  |  |
| 1 | 0 | 1 | 0 | 0 | 1 | 1 | 0 | 0 | 0 |  |  |  |  |
| 1 | 0 | 1 | 0 | 0 | 1 | 0 | 1 | 1 | 0 |  |  |  |  |
| 1 | 0 | 1 | 0 | 0 | 1 | 0 | 1 | 0 | 0 |  |  |  |  |
| 1 | 0 | 1 | 0 | 0 | 1 | 0 | 0 | 1 | 0 |  |  |  |  |
| 1 | 0 | 1 | 0 | 0 | 1 | 0 | 0 | 0 | 0 |  |  |  |  |
| 1 | 0 | 1 | 0 | 0 | 0 | 1 | 1 | 1 | 0 |  |  |  |  |
| 1 | 0 | 1 | 0 | 0 | 0 | 1 | 1 | 0 | 0 |  |  |  |  |
| 1 | 0 | 1 | 0 | 0 | 0 | 1 | 0 | 1 | 0 |  |  |  |  |
| 1 | 0 | 1 | 0 | 0 | 0 | 1 | 0 | 0 | 0 |  |  |  |  |
| 1 | 0 | 1 | 0 | 0 | 0 | 0 | 1 | 1 | 0 |  |  |  |  |
| 1 | 0 | 1 | 0 | 0 | 0 | 0 | 1 | 0 | 0 |  |  |  |  |
| 1 | 0 | 1 | 0 | 0 | 0 | 0 | 0 | 1 | 0 |  |  |  |  |
| 1 | 0 | 1 | 0 | 0 | 0 | 0 | 0 | 0 | 0 |  |  |  |  |
| 1 | 0 | 0 | 1 | 1 | 1 | 1 | 1 | 1 | 0 |  |  |  |  |
| 1 | 0 | 0 | 1 | 1 | 1 | 1 | 1 | 0 | 0 |  |  |  |  |
| 1 | 0 | 0 | 1 | 1 | 1 | 1 | 0 | 1 | 0 |  |  |  |  |
| 1 | 0 | 0 | 1 | 1 | 1 | 1 | 0 | 0 | 0 |  |  |  |  |
| 1 | 0 | 0 | 1 | 1 | 1 | 0 | 1 | 1 | 0 |  |  |  |  |
| 1 | 0 | 0 | 1 | 1 | 1 | 0 | 1 | 0 | 0 |  |  |  |  |
| 1 | 0 | 0 | 1 | 1 | 1 | 0 | 0 | 1 | 0 |  |  |  |  |
| 1 | 0 | 0 | 1 | 1 | 1 | 0 | 0 | 0 | 0 |  |  |  |  |
| 1 | 0 | 0 | 1 | 1 | 0 | 1 | 1 | 1 | 0 |  |  |  |  |
| 1 | 0 | 0 | 1 | 1 | 0 | 1 | 1 | 0 | 0 |  |  |  |  |
| 1 | 0 | 0 | 1 | 1 | 0 | 1 | 0 | 1 | 0 |  |  |  |  |
| 1 | 0 | 0 | 1 | 1 | 0 | 1 | 0 | 0 | 0 |  |  |  |  |
| 1 | 0 | 0 | 1 | 1 | 0 | 0 | 1 | 1 | 0 |  |  |  |  |
| 1 | 0 | 0 | 1 | 1 | 0 | 0 | 1 | 0 | 0 |  |  |  |  |
| 1 | 0 | 0 | 1 | 1 | 0 | 0 | 0 | 1 | 0 |  |  |  |  |
| 1 | 0 | 0 | 1 | 1 | 0 | 0 | 0 | 0 | 0 |  |  |  |  |
| 1 | 0 | 0 | 1 | 0 | 1 | 1 | 1 | 1 | 0 |  |  |  |  |
| 1 | 0 | 0 | 1 | 0 | 1 | 1 | 1 | 0 | 0 |  |  |  |  |
| 1 | 0 | 0 | 1 | 0 | 1 | 1 | 0 | 1 | 0 |  |  |  |  |
| 1 | 0 | 0 | 1 | 0 | 1 | 1 | 0 | 0 | 0 |  |  |  |  |
| 1 | 0 | 0 | 1 | 0 | 1 | 0 | 1 | 1 | 0 |  |  |  |  |
| 1 | 0 | 0 | 1 | 0 | 1 | 0 | 1 | 0 | 0 |  |  |  |  |
| 1 | 0 | 0 | 1 | 0 | 1 | 0 | 0 | 1 | 0 |  |  |  |  |
| 1 | 0 | 0 | 1 | 0 | 1 | 0 | 0 | 0 | 0 |  |  |  |  |
| 1 | 0 | 0 | 1 | 0 | 0 | 1 | 1 | 1 | 0 |  |  |  |  |
| 1 | 0 | 0 | 1 | 0 | 0 | 1 | 1 | 0 | 0 |  |  |  |  |
| 1 | 0 | 0 | 1 | 0 | 0 | 1 | 0 | 1 | 0 |  |  |  |  |
| 1 | 0 | 0 | 1 | 0 | 0 | 1 | 0 | 0 | 0 |  |  |  |  |
| 1 | 0 | 0 | 1 | 0 | 0 | 0 | 1 | 1 | 0 |  |  |  |  |
| 1 | 0 | 0 | 1 | 0 | 0 | 0 | 1 | 0 | 0 |  |  |  |  |
| 1 | 0 | 0 | 1 | 0 | 0 | 0 | 0 | 1 | 0 |  |  |  |  |
| 1 | 0 | 0 | 1 | 0 | 0 | 0 | 0 | 0 | 0 |  |  |  |  |
| 1 | 0 | 0 | 0 | 1 | 1 | 1 | 1 | 1 | 0 |  |  |  |  |
| 1 | 0 | 0 | 0 | 1 | 1 | 1 | 1 | 0 | 0 |  |  |  |  |
| 1 | 0 | 0 | 0 | 1 | 1 | 1 | 0 | 1 | 0 |  |  |  |  |
| 1 | 0 | 0 | 0 | 1 | 1 | 1 | 0 | 0 | 0 |  |  |  |  |
| 1 | 0 | 0 | 0 | 1 | 1 | 0 | 1 | 1 | 0 |  |  |  |  |
| 1 | 0 | 0 | 0 | 1 | 1 | 0 | 1 | 0 | 0 |  |  |  |  |
| 1 | 0 | 0 | 0 | 1 | 1 | 0 | 0 | 1 | 0 |  |  |  |  |
| 1 | 0 | 0 | 0 | 1 | 1 | 0 | 0 | 0 | 0 |  |  |  |  |
| 1 | 0 | 0 | 0 | 1 | 0 | 1 | 1 | 1 | 0 |  |  |  |  |
| 1 | 0 | 0 | 0 | 1 | 0 | 1 | 1 | 0 | 0 |  |  |  |  |
| 1 | 0 | 0 | 0 | 1 | 0 | 1 | 0 | 1 | 0 |  |  |  |  |
| 1 | 0 | 0 | 0 | 1 | 0 | 1 | 0 | 0 | 0 |  |  |  |  |
| 1 | 0 | 0 | 0 | 1 | 0 | 0 | 1 | 1 | 0 |  |  |  |  |
| 1 | 0 | 0 | 0 | 1 | 0 | 0 | 1 | 0 | 0 |  |  |  |  |
| 1 | 0 | 0 | 0 | 1 | 0 | 0 | 0 | 1 | 0 |  |  |  |  |
| 1 | 0 | 0 | 0 | 1 | 0 | 0 | 0 | 0 | 0 |  |  |  |  |
| 1 | 0 | 0 | 0 | 0 | 1 | 1 | 1 | 1 | 0 |  |  |  |  |
| 1 | 0 | 0 | 0 | 0 | 1 | 1 | 1 | 0 | 0 |  |  |  |  |
| 1 | 0 | 0 | 0 | 0 | 1 | 1 | 0 | 1 | 0 |  |  |  |  |
| 1 | 0 | 0 | 0 | 0 | 1 | 1 | 0 | 0 | 0 |  |  |  |  |
| 1 | 0 | 0 | 0 | 0 | 1 | 0 | 1 | 1 | 0 |  |  |  |  |
| 1 | 0 | 0 | 0 | 0 | 1 | 0 | 1 | 0 | 0 |  |  |  |  |
| 1 | 0 | 0 | 0 | 0 | 1 | 0 | 0 | 1 | 0 |  |  |  |  |
| 1 | 0 | 0 | 0 | 0 | 1 | 0 | 0 | 0 | 0 |  |  |  |  |
| 1 | 0 | 0 | 0 | 0 | 0 | 1 | 1 | 1 | 0 |  |  |  |  |
| 1 | 0 | 0 | 0 | 0 | 0 | 1 | 1 | 0 | 0 |  |  |  |  |
| 1 | 0 | 0 | 0 | 0 | 0 | 1 | 0 | 1 | 0 |  |  |  |  |
| 1 | 0 | 0 | 0 | 0 | 0 | 1 | 0 | 0 | 0 |  |  |  |  |
| 1 | 0 | 0 | 0 | 0 | 0 | 0 | 1 | 1 | 0 |  |  |  |  |
| 1 | 0 | 0 | 0 | 0 | 0 | 0 | 1 | 0 | 0 |  |  |  |  |
| 1 | 0 | 0 | 0 | 0 | 0 | 0 | 0 | 1 | 0 |  |  |  |  |
| 1 | 0 | 0 | 0 | 0 | 0 | 0 | 0 | 0 | 0 |  |  |  |  |
| 0 | 1 | 1 | 1 | 1 | 1 | 1 | 1 | 1 | 0 |  |  |  |  |
| 0 | 1 | 1 | 1 | 1 | 1 | 1 | 1 | 0 | 0 |  |  |  |  |
| 0 | 1 | 1 | 1 | 1 | 1 | 1 | 0 | 1 | 0 |  |  |  |  |
| 0 | 1 | 1 | 1 | 1 | 1 | 1 | 0 | 0 | 0 |  |  |  |  |
| 0 | 1 | 1 | 1 | 1 | 1 | 0 | 1 | 1 | 0 |  |  |  |  |
| 0 | 1 | 1 | 1 | 1 | 1 | 0 | 1 | 0 | 0 |  |  |  |  |
| 0 | 1 | 1 | 1 | 1 | 1 | 0 | 0 | 1 | 0 |  |  |  |  |
| 0 | 1 | 1 | 1 | 1 | 1 | 0 | 0 | 0 | 0 |  |  |  |  |
| 0 | 1 | 1 | 1 | 1 | 0 | 1 | 1 | 1 | 0 |  |  |  |  |
| 0 | 1 | 1 | 1 | 1 | 0 | 1 | 1 | 0 | 0 |  |  |  |  |
| 0 | 1 | 1 | 1 | 1 | 0 | 1 | 0 | 1 | 0 |  |  |  |  |
| 0 | 1 | 1 | 1 | 1 | 0 | 1 | 0 | 0 | 0 |  |  |  |  |
| 0 | 1 | 1 | 1 | 1 | 0 | 0 | 1 | 1 | 0 |  |  |  |  |
| 0 | 1 | 1 | 1 | 1 | 0 | 0 | 1 | 0 | 0 |  |  |  |  |
| 0 | 1 | 1 | 1 | 1 | 0 | 0 | 0 | 1 | 0 |  |  |  |  |
| 0 | 1 | 1 | 1 | 1 | 0 | 0 | 0 | 0 | 0 |  |  |  |  |
| 0 | 1 | 1 | 1 | 0 | 1 | 1 | 1 | 1 | 0 |  |  |  |  |
| 0 | 1 | 1 | 1 | 0 | 1 | 1 | 1 | 0 | 0 |  |  |  |  |
| 0 | 1 | 1 | 1 | 0 | 1 | 1 | 0 | 1 | 0 |  |  |  |  |
| 0 | 1 | 1 | 1 | 0 | 1 | 1 | 0 | 0 | 0 |  |  |  |  |
| 0 | 1 | 1 | 1 | 0 | 1 | 0 | 1 | 1 | 0 |  |  |  |  |
| 0 | 1 | 1 | 1 | 0 | 1 | 0 | 1 | 0 | 0 |  |  |  |  |
| 0 | 1 | 1 | 1 | 0 | 1 | 0 | 0 | 1 | 0 |  |  |  |  |
| 0 | 1 | 1 | 1 | 0 | 1 | 0 | 0 | 0 | 0 |  |  |  |  |
| 0 | 1 | 1 | 1 | 0 | 0 | 1 | 1 | 1 | 0 |  |  |  |  |
| 0 | 1 | 1 | 1 | 0 | 0 | 1 | 1 | 0 | 0 |  |  |  |  |
| 0 | 1 | 1 | 1 | 0 | 0 | 1 | 0 | 1 | 0 |  |  |  |  |
| 0 | 1 | 1 | 1 | 0 | 0 | 1 | 0 | 0 | 0 |  |  |  |  |
| 0 | 1 | 1 | 1 | 0 | 0 | 0 | 1 | 1 | 0 |  |  |  |  |
| 0 | 1 | 1 | 1 | 0 | 0 | 0 | 1 | 0 | 0 |  |  |  |  |
| 0 | 1 | 1 | 1 | 0 | 0 | 0 | 0 | 1 | 0 |  |  |  |  |
| 0 | 1 | 1 | 1 | 0 | 0 | 0 | 0 | 0 | 0 |  |  |  |  |
| 0 | 1 | 1 | 0 | 1 | 1 | 1 | 1 | 1 | 0 |  |  |  |  |
| 0 | 1 | 1 | 0 | 1 | 1 | 1 | 1 | 0 | 0 |  |  |  |  |
| 0 | 1 | 1 | 0 | 1 | 1 | 1 | 0 | 1 | 0 |  |  |  |  |
| 0 | 1 | 1 | 0 | 1 | 1 | 1 | 0 | 0 | 0 |  |  |  |  |
| 0 | 1 | 1 | 0 | 1 | 1 | 0 | 1 | 1 | 0 |  |  |  |  |
| 0 | 1 | 1 | 0 | 1 | 1 | 0 | 1 | 0 | 0 |  |  |  |  |
| 0 | 1 | 1 | 0 | 1 | 1 | 0 | 0 | 1 | 0 |  |  |  |  |
| 0 | 1 | 1 | 0 | 1 | 1 | 0 | 0 | 0 | 0 |  |  |  |  |
| 0 | 1 | 1 | 0 | 1 | 0 | 1 | 1 | 1 | 0 |  |  |  |  |
| 0 | 1 | 1 | 0 | 1 | 0 | 1 | 1 | 0 | 0 |  |  |  |  |
| 0 | 1 | 1 | 0 | 1 | 0 | 1 | 0 | 1 | 0 |  |  |  |  |
| 0 | 1 | 1 | 0 | 1 | 0 | 1 | 0 | 0 | 0 |  |  |  |  |
| 0 | 1 | 1 | 0 | 1 | 0 | 0 | 1 | 1 | 0 |  |  |  |  |
| 0 | 1 | 1 | 0 | 1 | 0 | 0 | 1 | 0 | 0 |  |  |  |  |
| 0 | 1 | 1 | 0 | 1 | 0 | 0 | 0 | 1 | 0 |  |  |  |  |
| 0 | 1 | 1 | 0 | 1 | 0 | 0 | 0 | 0 | 0 |  |  |  |  |
| 0 | 1 | 1 | 0 | 0 | 1 | 1 | 1 | 1 | 0 |  |  |  |  |
| 0 | 1 | 1 | 0 | 0 | 1 | 1 | 1 | 0 | 0 |  |  |  |  |
| 0 | 1 | 1 | 0 | 0 | 1 | 1 | 0 | 1 | 0 |  |  |  |  |
| 0 | 1 | 1 | 0 | 0 | 1 | 1 | 0 | 0 | 0 |  |  |  |  |
| 0 | 1 | 1 | 0 | 0 | 1 | 0 | 1 | 1 | 0 |  |  |  |  |
| 0 | 1 | 1 | 0 | 0 | 1 | 0 | 1 | 0 | 0 |  |  |  |  |
| 0 | 1 | 1 | 0 | 0 | 1 | 0 | 0 | 1 | 0 |  |  |  |  |
| 0 | 1 | 1 | 0 | 0 | 1 | 0 | 0 | 0 | 0 |  |  |  |  |
| 0 | 1 | 1 | 0 | 0 | 0 | 1 | 1 | 1 | 0 |  |  |  |  |
| 0 | 1 | 1 | 0 | 0 | 0 | 1 | 1 | 0 | 0 |  |  |  |  |
| 0 | 1 | 1 | 0 | 0 | 0 | 1 | 0 | 1 | 0 |  |  |  |  |
| 0 | 1 | 1 | 0 | 0 | 0 | 1 | 0 | 0 | 0 |  |  |  |  |
| 0 | 1 | 1 | 0 | 0 | 0 | 0 | 1 | 1 | 0 |  |  |  |  |
| 0 | 1 | 1 | 0 | 0 | 0 | 0 | 1 | 0 | 0 |  |  |  |  |
| 0 | 1 | 1 | 0 | 0 | 0 | 0 | 0 | 1 | 0 |  |  |  |  |
| 0 | 1 | 1 | 0 | 0 | 0 | 0 | 0 | 0 | 0 |  |  |  |  |
| 0 | 1 | 0 | 1 | 1 | 1 | 1 | 1 | 1 | 0 |  |  |  |  |
| 0 | 1 | 0 | 1 | 1 | 1 | 1 | 1 | 0 | 0 |  |  |  |  |
| 0 | 1 | 0 | 1 | 1 | 1 | 1 | 0 | 1 | 0 |  |  |  |  |
| 0 | 1 | 0 | 1 | 1 | 1 | 1 | 0 | 0 | 0 |  |  |  |  |
| 0 | 1 | 0 | 1 | 1 | 1 | 0 | 1 | 1 | 0 |  |  |  |  |
| 0 | 1 | 0 | 1 | 1 | 1 | 0 | 1 | 0 | 0 |  |  |  |  |
| 0 | 1 | 0 | 1 | 1 | 1 | 0 | 0 | 1 | 0 |  |  |  |  |
| 0 | 1 | 0 | 1 | 1 | 1 | 0 | 0 | 0 | 0 |  |  |  |  |
| 0 | 1 | 0 | 1 | 1 | 0 | 1 | 1 | 1 | 0 |  |  |  |  |
| 0 | 1 | 0 | 1 | 1 | 0 | 1 | 1 | 0 | 0 |  |  |  |  |
| 0 | 1 | 0 | 1 | 1 | 0 | 1 | 0 | 1 | 0 |  |  |  |  |
| 0 | 1 | 0 | 1 | 1 | 0 | 1 | 0 | 0 | 0 |  |  |  |  |
| 0 | 1 | 0 | 1 | 1 | 0 | 0 | 1 | 1 | 0 |  |  |  |  |
| 0 | 1 | 0 | 1 | 1 | 0 | 0 | 1 | 0 | 0 |  |  |  |  |
| 0 | 1 | 0 | 1 | 1 | 0 | 0 | 0 | 1 | 0 |  |  |  |  |
| 0 | 1 | 0 | 1 | 1 | 0 | 0 | 0 | 0 | 0 |  |  |  |  |
| 0 | 1 | 0 | 1 | 0 | 1 | 1 | 1 | 1 | 0 |  |  |  |  |
| 0 | 1 | 0 | 1 | 0 | 1 | 1 | 1 | 0 | 0 |  |  |  |  |
| 0 | 1 | 0 | 1 | 0 | 1 | 1 | 0 | 1 | 0 |  |  |  |  |
| 0 | 1 | 0 | 1 | 0 | 1 | 1 | 0 | 0 | 0 |  |  |  |  |
| 0 | 1 | 0 | 1 | 0 | 1 | 0 | 1 | 1 | 0 |  |  |  |  |
| 0 | 1 | 0 | 1 | 0 | 1 | 0 | 1 | 0 | 0 |  |  |  |  |
| 0 | 1 | 0 | 1 | 0 | 1 | 0 | 0 | 1 | 0 |  |  |  |  |
| 0 | 1 | 0 | 1 | 0 | 1 | 0 | 0 | 0 | 0 |  |  |  |  |
| 0 | 1 | 0 | 1 | 0 | 0 | 1 | 1 | 1 | 0 |  |  |  |  |
| 0 | 1 | 0 | 1 | 0 | 0 | 1 | 1 | 0 | 0 |  |  |  |  |
| 0 | 1 | 0 | 1 | 0 | 0 | 1 | 0 | 1 | 0 |  |  |  |  |
| 0 | 1 | 0 | 1 | 0 | 0 | 1 | 0 | 0 | 0 |  |  |  |  |
| 0 | 1 | 0 | 1 | 0 | 0 | 0 | 1 | 1 | 0 |  |  |  |  |
| 0 | 1 | 0 | 1 | 0 | 0 | 0 | 1 | 0 | 0 |  |  |  |  |
| 0 | 1 | 0 | 1 | 0 | 0 | 0 | 0 | 1 | 0 |  |  |  |  |
| 0 | 1 | 0 | 1 | 0 | 0 | 0 | 0 | 0 | 0 |  |  |  |  |
| 0 | 1 | 0 | 0 | 1 | 1 | 1 | 1 | 1 | 0 |  |  |  |  |
| 0 | 1 | 0 | 0 | 1 | 1 | 1 | 1 | 0 | 0 |  |  |  |  |
| 0 | 1 | 0 | 0 | 1 | 1 | 1 | 0 | 1 | 0 |  |  |  |  |
| 0 | 1 | 0 | 0 | 1 | 1 | 1 | 0 | 0 | 0 |  |  |  |  |
| 0 | 1 | 0 | 0 | 1 | 1 | 0 | 1 | 1 | 0 |  |  |  |  |
| 0 | 1 | 0 | 0 | 1 | 1 | 0 | 1 | 0 | 0 |  |  |  |  |
| 0 | 1 | 0 | 0 | 1 | 1 | 0 | 0 | 1 | 0 |  |  |  |  |
| 0 | 1 | 0 | 0 | 1 | 1 | 0 | 0 | 0 | 0 |  |  |  |  |
| 0 | 1 | 0 | 0 | 1 | 0 | 1 | 1 | 1 | 0 |  |  |  |  |
| 0 | 1 | 0 | 0 | 1 | 0 | 1 | 1 | 0 | 0 |  |  |  |  |
| 0 | 1 | 0 | 0 | 1 | 0 | 1 | 0 | 1 | 0 |  |  |  |  |
| 0 | 1 | 0 | 0 | 1 | 0 | 1 | 0 | 0 | 0 |  |  |  |  |
| 0 | 1 | 0 | 0 | 1 | 0 | 0 | 1 | 1 | 0 |  |  |  |  |
| 0 | 1 | 0 | 0 | 1 | 0 | 0 | 1 | 0 | 0 |  |  |  |  |
| 0 | 1 | 0 | 0 | 1 | 0 | 0 | 0 | 1 | 0 |  |  |  |  |
| 0 | 1 | 0 | 0 | 1 | 0 | 0 | 0 | 0 | 0 |  |  |  |  |
| 0 | 1 | 0 | 0 | 0 | 1 | 1 | 1 | 1 | 0 |  |  |  |  |
| 0 | 1 | 0 | 0 | 0 | 1 | 1 | 1 | 0 | 0 |  |  |  |  |
| 0 | 1 | 0 | 0 | 0 | 1 | 1 | 0 | 1 | 0 |  |  |  |  |
| 0 | 1 | 0 | 0 | 0 | 1 | 1 | 0 | 0 | 0 |  |  |  |  |
| 0 | 1 | 0 | 0 | 0 | 1 | 0 | 1 | 1 | 0 |  |  |  |  |
| 0 | 1 | 0 | 0 | 0 | 1 | 0 | 1 | 0 | 0 |  |  |  |  |
| 0 | 1 | 0 | 0 | 0 | 1 | 0 | 0 | 1 | 0 |  |  |  |  |
| 0 | 1 | 0 | 0 | 0 | 1 | 0 | 0 | 0 | 0 |  |  |  |  |
| 0 | 1 | 0 | 0 | 0 | 0 | 1 | 1 | 1 | 0 |  |  |  |  |
| 0 | 1 | 0 | 0 | 0 | 0 | 1 | 1 | 0 | 0 |  |  |  |  |
| 0 | 1 | 0 | 0 | 0 | 0 | 1 | 0 | 1 | 0 |  |  |  |  |
| 0 | 1 | 0 | 0 | 0 | 0 | 1 | 0 | 0 | 0 |  |  |  |  |
| 0 | 1 | 0 | 0 | 0 | 0 | 0 | 1 | 1 | 0 |  |  |  |  |
| 0 | 1 | 0 | 0 | 0 | 0 | 0 | 1 | 0 | 0 |  |  |  |  |
| 0 | 1 | 0 | 0 | 0 | 0 | 0 | 0 | 1 | 0 |  |  |  |  |
| 0 | 1 | 0 | 0 | 0 | 0 | 0 | 0 | 0 | 0 |  |  |  |  |
| 0 | 0 | 1 | 1 | 1 | 1 | 1 | 1 | 1 | 0 |  |  |  |  |
| 0 | 0 | 1 | 1 | 1 | 1 | 1 | 1 | 0 | 0 |  |  |  |  |
| 0 | 0 | 1 | 1 | 1 | 1 | 1 | 0 | 1 | 0 |  |  |  |  |
| 0 | 0 | 1 | 1 | 1 | 1 | 1 | 0 | 0 | 0 |  |  |  |  |
| 0 | 0 | 1 | 1 | 1 | 1 | 0 | 1 | 1 | 0 |  |  |  |  |
| 0 | 0 | 1 | 1 | 1 | 1 | 0 | 1 | 0 | 0 |  |  |  |  |
| 0 | 0 | 1 | 1 | 1 | 1 | 0 | 0 | 1 | 0 |  |  |  |  |
| 0 | 0 | 1 | 1 | 1 | 1 | 0 | 0 | 0 | 0 |  |  |  |  |
| 0 | 0 | 1 | 1 | 1 | 0 | 1 | 1 | 1 | 0 |  |  |  |  |
| 0 | 0 | 1 | 1 | 1 | 0 | 1 | 1 | 0 | 0 |  |  |  |  |
| 0 | 0 | 1 | 1 | 1 | 0 | 1 | 0 | 1 | 0 |  |  |  |  |
| 0 | 0 | 1 | 1 | 1 | 0 | 1 | 0 | 0 | 0 |  |  |  |  |
| 0 | 0 | 1 | 1 | 1 | 0 | 0 | 1 | 1 | 0 |  |  |  |  |
| 0 | 0 | 1 | 1 | 1 | 0 | 0 | 1 | 0 | 0 |  |  |  |  |
| 0 | 0 | 1 | 1 | 1 | 0 | 0 | 0 | 1 | 0 |  |  |  |  |
| 0 | 0 | 1 | 1 | 1 | 0 | 0 | 0 | 0 | 0 |  |  |  |  |
| 0 | 0 | 1 | 1 | 0 | 1 | 1 | 1 | 1 | 0 |  |  |  |  |
| 0 | 0 | 1 | 1 | 0 | 1 | 1 | 1 | 0 | 0 |  |  |  |  |
| 0 | 0 | 1 | 1 | 0 | 1 | 1 | 0 | 1 | 0 |  |  |  |  |
| 0 | 0 | 1 | 1 | 0 | 1 | 1 | 0 | 0 | 0 |  |  |  |  |
| 0 | 0 | 1 | 1 | 0 | 1 | 0 | 1 | 0 | 0 |  |  |  |  |
| 0 | 0 | 1 | 1 | 0 | 1 | 0 | 0 | 1 | 0 |  |  |  |  |
| 0 | 0 | 1 | 1 | 0 | 1 | 0 | 0 | 0 | 0 |  |  |  |  |
| 0 | 0 | 1 | 1 | 0 | 0 | 1 | 1 | 1 | 0 |  |  |  |  |
| 0 | 0 | 1 | 1 | 0 | 0 | 1 | 1 | 0 | 0 |  |  |  |  |
| 0 | 0 | 1 | 1 | 0 | 0 | 1 | 0 | 1 | 0 |  |  |  |  |
| 0 | 0 | 1 | 1 | 0 | 0 | 1 | 0 | 0 | 0 |  |  |  |  |
| 0 | 0 | 1 | 1 | 0 | 0 | 0 | 1 | 1 | 0 |  |  |  |  |
| 0 | 0 | 1 | 1 | 0 | 0 | 0 | 1 | 0 | 0 |  |  |  |  |
| 0 | 0 | 1 | 1 | 0 | 0 | 0 | 0 | 1 | 0 |  |  |  |  |
| 0 | 0 | 1 | 1 | 0 | 0 | 0 | 0 | 0 | 0 |  |  |  |  |
| 0 | 0 | 1 | 0 | 1 | 1 | 1 | 1 | 1 | 0 |  |  |  |  |
| 0 | 0 | 1 | 0 | 1 | 1 | 1 | 1 | 0 | 0 |  |  |  |  |
| 0 | 0 | 1 | 0 | 1 | 1 | 1 | 0 | 1 | 0 |  |  |  |  |
| 0 | 0 | 1 | 0 | 1 | 1 | 1 | 0 | 0 | 0 |  |  |  |  |
| 0 | 0 | 1 | 0 | 1 | 1 | 0 | 1 | 1 | 0 |  |  |  |  |
| 0 | 0 | 1 | 0 | 1 | 1 | 0 | 1 | 0 | 0 |  |  |  |  |
| 0 | 0 | 1 | 0 | 1 | 1 | 0 | 0 | 1 | 0 |  |  |  |  |
| 0 | 0 | 1 | 0 | 1 | 1 | 0 | 0 | 0 | 0 |  |  |  |  |
| 0 | 0 | 1 | 0 | 1 | 0 | 1 | 1 | 1 | 0 |  |  |  |  |
| 0 | 0 | 1 | 0 | 1 | 0 | 1 | 1 | 0 | 0 |  |  |  |  |
| 0 | 0 | 1 | 0 | 1 | 0 | 1 | 0 | 1 | 0 |  |  |  |  |
| 0 | 0 | 1 | 0 | 1 | 0 | 1 | 0 | 0 | 0 |  |  |  |  |
| 0 | 0 | 1 | 0 | 1 | 0 | 0 | 1 | 1 | 0 |  |  |  |  |
| 0 | 0 | 1 | 0 | 1 | 0 | 0 | 1 | 0 | 0 |  |  |  |  |
| 0 | 0 | 1 | 0 | 1 | 0 | 0 | 0 | 1 | 0 |  |  |  |  |
| 0 | 0 | 1 | 0 | 1 | 0 | 0 | 0 | 0 | 0 |  |  |  |  |
| 0 | 0 | 1 | 0 | 0 | 1 | 1 | 1 | 1 | 0 |  |  |  |  |
| 0 | 0 | 1 | 0 | 0 | 1 | 1 | 1 | 0 | 0 |  |  |  |  |
| 0 | 0 | 1 | 0 | 0 | 1 | 1 | 0 | 1 | 0 |  |  |  |  |
| 0 | 0 | 1 | 0 | 0 | 1 | 1 | 0 | 0 | 0 |  |  |  |  |
| 0 | 0 | 1 | 0 | 0 | 1 | 0 | 1 | 1 | 0 |  |  |  |  |
| 0 | 0 | 1 | 0 | 0 | 1 | 0 | 1 | 0 | 0 |  |  |  |  |
| 0 | 0 | 1 | 0 | 0 | 1 | 0 | 0 | 1 | 0 |  |  |  |  |
| 0 | 0 | 1 | 0 | 0 | 1 | 0 | 0 | 0 | 0 |  |  |  |  |
| 0 | 0 | 1 | 0 | 0 | 0 | 1 | 1 | 1 | 0 |  |  |  |  |
| 0 | 0 | 1 | 0 | 0 | 0 | 1 | 1 | 0 | 0 |  |  |  |  |
| 0 | 0 | 1 | 0 | 0 | 0 | 1 | 0 | 1 | 0 |  |  |  |  |
| 0 | 0 | 1 | 0 | 0 | 0 | 1 | 0 | 0 | 0 |  |  |  |  |
| 0 | 0 | 1 | 0 | 0 | 0 | 0 | 1 | 0 | 0 |  |  |  |  |
| 0 | 0 | 1 | 0 | 0 | 0 | 0 | 0 | 1 | 0 |  |  |  |  |
| 0 | 0 | 1 | 0 | 0 | 0 | 0 | 0 | 0 | 0 |  |  |  |  |
| 0 | 0 | 0 | 1 | 1 | 1 | 1 | 1 | 1 | 0 |  |  |  |  |
| 0 | 0 | 0 | 1 | 1 | 1 | 1 | 1 | 0 | 0 |  |  |  |  |
| 0 | 0 | 0 | 1 | 1 | 1 | 1 | 0 | 1 | 0 |  |  |  |  |
| 0 | 0 | 0 | 1 | 1 | 1 | 1 | 0 | 0 | 0 |  |  |  |  |
| 0 | 0 | 0 | 1 | 1 | 1 | 0 | 1 | 1 | 0 |  |  |  |  |
| 0 | 0 | 0 | 1 | 1 | 1 | 0 | 1 | 0 | 0 |  |  |  |  |
| 0 | 0 | 0 | 1 | 1 | 1 | 0 | 0 | 1 | 0 |  |  |  |  |
| 0 | 0 | 0 | 1 | 1 | 1 | 0 | 0 | 0 | 0 |  |  |  |  |
| 0 | 0 | 0 | 1 | 1 | 0 | 1 | 1 | 1 | 0 |  |  |  |  |
| 0 | 0 | 0 | 1 | 1 | 0 | 1 | 1 | 0 | 0 |  |  |  |  |
| 0 | 0 | 0 | 1 | 1 | 0 | 1 | 0 | 1 | 0 |  |  |  |  |
| 0 | 0 | 0 | 1 | 1 | 0 | 1 | 0 | 0 | 0 |  |  |  |  |
| 0 | 0 | 0 | 1 | 1 | 0 | 0 | 1 | 1 | 0 |  |  |  |  |
| 0 | 0 | 0 | 1 | 1 | 0 | 0 | 1 | 0 | 0 |  |  |  |  |
| 0 | 0 | 0 | 1 | 1 | 0 | 0 | 0 | 1 | 0 |  |  |  |  |
| 0 | 0 | 0 | 1 | 1 | 0 | 0 | 0 | 0 | 0 |  |  |  |  |
| 0 | 0 | 0 | 1 | 0 | 1 | 1 | 1 | 1 | 0 |  |  |  |  |
| 0 | 0 | 0 | 1 | 0 | 1 | 1 | 1 | 0 | 0 |  |  |  |  |
| 0 | 0 | 0 | 1 | 0 | 1 | 1 | 0 | 1 | 0 |  |  |  |  |
| 0 | 0 | 0 | 1 | 0 | 1 | 1 | 0 | 0 | 0 |  |  |  |  |
| 0 | 0 | 0 | 1 | 0 | 1 | 0 | 1 | 1 | 0 |  |  |  |  |
| 0 | 0 | 0 | 1 | 0 | 1 | 0 | 1 | 0 | 0 |  |  |  |  |
| 0 | 0 | 0 | 1 | 0 | 1 | 0 | 0 | 1 | 0 |  |  |  |  |
| 0 | 0 | 0 | 1 | 0 | 1 | 0 | 0 | 0 | 0 |  |  |  |  |
| 0 | 0 | 0 | 1 | 0 | 0 | 1 | 1 | 1 | 0 |  |  |  |  |
| 0 | 0 | 0 | 1 | 0 | 0 | 1 | 1 | 0 | 0 |  |  |  |  |
| 0 | 0 | 0 | 1 | 0 | 0 | 1 | 0 | 1 | 0 |  |  |  |  |
| 0 | 0 | 0 | 1 | 0 | 0 | 1 | 0 | 0 | 0 |  |  |  |  |
| 0 | 0 | 0 | 1 | 0 | 0 | 0 | 1 | 1 | 0 |  |  |  |  |
| 0 | 0 | 0 | 1 | 0 | 0 | 0 | 1 | 0 | 0 |  |  |  |  |
| 0 | 0 | 0 | 1 | 0 | 0 | 0 | 0 | 1 | 0 |  |  |  |  |
| 0 | 0 | 0 | 1 | 0 | 0 | 0 | 0 | 0 | 0 |  |  |  |  |
| 0 | 0 | 0 | 0 | 1 | 1 | 1 | 1 | 1 | 0 |  |  |  |  |
| 0 | 0 | 0 | 0 | 1 | 1 | 1 | 1 | 0 | 0 |  |  |  |  |
| 0 | 0 | 0 | 0 | 1 | 1 | 1 | 0 | 1 | 0 |  |  |  |  |
| 0 | 0 | 0 | 0 | 1 | 1 | 1 | 0 | 0 | 0 |  |  |  |  |
| 0 | 0 | 0 | 0 | 1 | 1 | 0 | 1 | 1 | 0 |  |  |  |  |
| 0 | 0 | 0 | 0 | 1 | 1 | 0 | 1 | 0 | 0 |  |  |  |  |
| 0 | 0 | 0 | 0 | 1 | 1 | 0 | 0 | 1 | 0 |  |  |  |  |
| 0 | 0 | 0 | 0 | 1 | 1 | 0 | 0 | 0 | 0 |  |  |  |  |
| 0 | 0 | 0 | 0 | 1 | 0 | 1 | 1 | 1 | 0 |  |  |  |  |
| 0 | 0 | 0 | 0 | 1 | 0 | 1 | 1 | 0 | 0 |  |  |  |  |
| 0 | 0 | 0 | 0 | 1 | 0 | 1 | 0 | 1 | 0 |  |  |  |  |
| 0 | 0 | 0 | 0 | 1 | 0 | 1 | 0 | 0 | 0 |  |  |  |  |
| 0 | 0 | 0 | 0 | 1 | 0 | 0 | 1 | 1 | 0 |  |  |  |  |
| 0 | 0 | 0 | 0 | 1 | 0 | 0 | 1 | 0 | 0 |  |  |  |  |
| 0 | 0 | 0 | 0 | 1 | 0 | 0 | 0 | 1 | 0 |  |  |  |  |
| 0 | 0 | 0 | 0 | 1 | 0 | 0 | 0 | 0 | 0 |  |  |  |  |
| 0 | 0 | 0 | 0 | 0 | 1 | 1 | 1 | 1 | 0 |  |  |  |  |
| 0 | 0 | 0 | 0 | 0 | 1 | 1 | 1 | 0 | 0 |  |  |  |  |
| 0 | 0 | 0 | 0 | 0 | 1 | 1 | 0 | 1 | 0 |  |  |  |  |
| 0 | 0 | 0 | 0 | 0 | 1 | 1 | 0 | 0 | 0 |  |  |  |  |
| 0 | 0 | 0 | 0 | 0 | 1 | 0 | 1 | 1 | 0 |  |  |  |  |
| 0 | 0 | 0 | 0 | 0 | 1 | 0 | 1 | 0 | 0 |  |  |  |  |
| 0 | 0 | 0 | 0 | 0 | 1 | 0 | 0 | 1 | 0 |  |  |  |  |
| 0 | 0 | 0 | 0 | 0 | 1 | 0 | 0 | 0 | 0 |  |  |  |  |
| 0 | 0 | 0 | 0 | 0 | 0 | 1 | 1 | 1 | 0 |  |  |  |  |
| 0 | 0 | 0 | 0 | 0 | 0 | 1 | 1 | 0 | 0 |  |  |  |  |
| 0 | 0 | 0 | 0 | 0 | 0 | 1 | 0 | 1 | 0 |  |  |  |  |
| 0 | 0 | 0 | 0 | 0 | 0 | 1 | 0 | 0 | 0 |  |  |  |  |
| 0 | 0 | 0 | 0 | 0 | 0 | 0 | 1 | 1 | 0 |  |  |  |  |
| 0 | 0 | 0 | 0 | 0 | 0 | 0 | 1 | 0 | 0 |  |  |  |  |
| 0 | 0 | 0 | 0 | 0 | 0 | 0 | 0 | 1 | 0 |  |  |  |  |
| 0 | 0 | 0 | 0 | 0 | 0 | 0 | 0 | 0 | 0 |  |  |  |  |
